# A vicious circle in breast cancer: The interplay between inflammation, reactive oxygen species, and microRNAs

**DOI:** 10.3389/fonc.2022.980694

**Published:** 2022-09-26

**Authors:** Valeria Villarreal-García, José Roberto Estupiñan-Jiménez, Pablo E. Vivas-Mejía, Vianey Gonzalez-Villasana, José Manuel Vázquez-Guillén, Diana Reséndez-Pérez

**Affiliations:** ^1^ Departmento de Biología Celular y Genética, Facultad de Ciencias Biológicas, Universidad Autónoma de Nuevo León, San Nicolás de los Garza, Nuevo León, Mexico; ^2^ Department of Biochemestry, Medical Sciences Campus, University of Puerto Rico, San Juan, Puerto Rico; ^3^ Comprehensive Cancer Center, Medical Sciences Campus, University of Puerto Rico, San Juan, Puerto Rico; ^4^ Departamento de Inmunología y Virología, Facultad de Ciencias Biológicas, Universidad Autónoma de Nuevo León, San Nicolás de los Garza, Nuevo León, Mexico

**Keywords:** inflammation, ros, microRNAs, breast cancer, regulation

## Abstract

Breast cancer (BC) is the most common cancer in women worldwide. This highly heterogeneous disease is molecularly stratified into luminal A, luminal B, HER2, triple-negative/basal-like, and normal-like subtypes. An important aspect in BC progression is the activation of inflammatory processes. The activation of CD8+/Th1, NK, and M1 tumor associated macrophages (TAMs), leads to tumor destruction. In contrast, an anti-inflammatory response mediated by CD4+/Th2 and M2 TAMs will favor tumor progression. Inflammation also stimulates the production of inflammatory mediators like reactive oxygen species (ROS). In chronic inflammation, ROS activates oxidative stress and endothelial dysfunction. In cancer, ROS plays a dual role with anti-tumorigenic and pro-tumorigenic effects in cell signaling pathways that control proliferation, survival, apoptosis, and inflammation. MicroRNAs (miRNAs), which are known to be involved in BC progression and inflammation, can be regulated by ROS. At the same time, miRNAs regulate the expression of genes modulating oxidative stress. In this review, we will discuss the interplay between inflammation, ROS, and miRNAs as anticancer and tumor promoter molecules in BC. A clear understanding of the role of miRNAs in the regulation of ROS production and inflammation, may lead to new opportunities for therapy in BC.

## Introduction

In women, breast cancer (BC) is the most diagnosed cancer with an estimated incidence of 2.3 million new cases (11.7% of all cancer cases) and it is the leading global cause of cancer since 2020. Although the mortality of BC patients has decreased in the last decade, it remains the leading cause of cancer death in women, and it is the fifth leading cause of cancer mortality worldwide, with around 685,000 deaths per year (1). Most BC are sporadic (~90%), and their increasing incidence has been related to the alteration of risk factors such as prolonged exposure to endogenous estrogens, age, sedentary lifestyle, alcohol consumption, obesity, exposure to ionizing radiation, and hormone replacement therapy (especially the combination of estrogens and progestogens) (2, 3).

The majority of BCs start in the lobes or in the ducts that connect the lobes to the nipple (4). The mammary epithelial cells acquire the ability to grow “abnormally” for years while remaining within the ducts or breast lobes (5). Once leaving the ducts or lobes, cancerous cells can metastasize through the blood or lymphatic system to distant organs such as the lungs, liver, brain or bones. During metastasis, cell migration and invasion occurs through a cascade of molecular events directed by genetic mutations and altered gene expression (6).

Inflammation is a mechanism of the immune system that responds to external or internal stimuli, removes the aggressor, and restores homeostasis. When chronic inflammation appears, diseases can develop, including BC (7). BC progression requires the participation of growth signaling pathways, as well as supportive signals from immune cells, proinflammatory cytokines, and growth factors present in an inflammatory tumor microenvironment (TME) (8–10) are necessary. Additionally, inflammation is important in cancer development and metastasis (11). Inflammation also promotes the production of reactive oxygen species (ROS), which are free radicals, ions, or molecules with a single unpaired electron (12). ROS have a dual role, they can act beneficially as signaling molecules when they are produced at low levels, but when ROS is elevated, they can induce damage, resulting in genetic instability and tumorigenesis (13–15). ROS can influence different processes that induce cancer progression, including BC, in association with the inflammatory TME (16).

ROS participate in complexes regulatory pathways with other molecules involved in inflammation and oncological processes including the microRNA (miRNAs) (17). MiRNAs are small non-coding RNA molecules that participate in gene regulation (18). Their mechanism of action depends on the complementarity of base pairs with the target messenger RNA (mRNA): if it is total, a degradation of the mRNA occurs; if it is partial, it causes a decrease in mRNA translation (19). The deregulation of miRNAs has been reported in multiple types of cancer, including BC, and has been associated with the progression of this disease (20). Interestingly, miRNA deregulation has a direct connection with ROS regulation, and ROS levels are also key in the regulation of multiple miRNAs, highlighting the connection between the regulatory networks of both molecules in BC (21, 22). A better understanding of the relationship between both molecules will allow the development of novel therapeutic approaches.

## Breast cancer subtypes

BC is a highly heterogeneous disease with molecular, biological, and morphological variations, and thus has different behavior and responses to treatment. BC classification is based on its histological and molecular features, which can be used for prognosis or to predict treatment response and overall survival (23, 24). Histologic classification evaluates different parameters like tumor cell type, extracellular secretions, structural features, immunohistochemical profile as well as the pathological growth pattern and anatomical origin (25, 26). The histological differentiation is a good prognostic factor and it is used to make better clinical decisions (27). TNM staging, on the other hand, uses clinical and pathologic information of tumor size (T), the status of regional lymph nodes (N), and distant metastases (M). The staging combines these factors and stratifies the disease into five stages (0, I, II, III, and IV) (28).

Modern genomic techniques have confirmed an association between the natural history of BC with a particular gene expression profile of the tumor (29). This molecular classification is more effective than the anatomical criteria, and allows for prognosis and treatment options to be defined individually (30). Under this classification BC is divided into five molecular subtypes according to a combination of gene expression profiles and immunochemistry information: luminal A, luminal B, human epidermal growth factor receptor 2 (HER2), basal-like BC, and normal-like tumors. This molecular classification is based on the presence of hormone receptors and HER2 status (**Table 1**) (31).

**Table 1:** Breast Cancer Molecular Subtypes.

Subtype	IHC Phenotype	Prognosis	Characteristics	References
**Luminal A**	ER+, PR ≥ 20%, HER2-, Ki67 low	Good	Most common subtype Lower-grade tumor Diagnosed at early stages	(31–33)
**Luminal B**	ER+, PR < 20%, HER2+/-, Ki67 high	Intermediate	Higher grade tumor	(31–33)
**HER2**	ER-, PR-, HER2+, Ki67 high	Poor	Less common subtype Higher grade tumor Highly aggressive	(31–34)
**Basal like**	ER-, PR-, HER2-, Ki67 high	Poor	Most aggressive subtype High invasiveness High metastatic potential	(31–33, 35)
**Normal-like**	ER+, PR+, HER2-, Ki67 low	Intermediate	Resembling the normal breast profiling	(36)

ER, estrogen receptor; PR, progesterone receptor; HER2, human epidermal growth factor receptor 2; Ki67, proliferation index.

Luminal breast cancers are associated with estrogen receptor (ER) activation and they are divided into two subgroups according to the expression of proliferation-related genes (37). Luminal A is the most common molecular subtype, and it is considered of low grade, with the best prognosis among all subtypes. It has positive hormone receptors, like ER and progesterone receptor (PR), and negative HER2, as well as a low expression of proliferation-related genes (38, 39). In contrast, luminal B tumors tend to be of higher grade and have a worse prognosis, lower expression of ER-related genes, variable HER2 expression, and high expression of proliferation-related genes (40). Because of their characteristics, luminal A tumors show a good response to hormonal therapy, whereas luminal B tumors may be candidates for chemotherapy because of their high proliferation rates (23, 31). The Ki67 index, also known as a proliferation biomarker, is an independent prognostic factor in BC and it is used to evaluate the risk of recurrence and BC survival; higher Ki67 correlates with tumor grade and poor prognosis (32, 41–43). This marker is also used to differentiate luminal A from B tumors (44).

ER-negative cancers are also divided into two subgroups: HER2-enriched and basal-like/triple negative BC. The HER2-enriched group is distinguished by HER2 overexpression and genes associated with HER2 signaling (37). These HER2-overexpressing tumors are generally high-grade, ER-, PR-, and are clinically aggressive. However, they are very sensitive to anti-HER2 targeted therapy. Most tumors within this subgroup are HER2+, but a small number of HER2+ cancers co-express ER and are classified as luminal B (39, 45).

Basal-like BC is associated with high expression of high-molecular-weight cytokeratins, P-cadherin, and the epidermal growth factor receptor in normal myoepithelial and basal mammary cells (39). This subtype of BC also exhibits overexpression of genes related with proliferation, but they do not express ER, PR or HER2. Histologically, they are high grade, with a high proliferation index, and a triple negative phenotype (45). BC that carries the *BRCA1* mutation mostly belong to the basal-like subtype. Although, the terms basal-like and triple negative BC have been used indistinctly, not all triple negative breast cancers are of the basal-like subtype (23). Triple negative BC has different molecular features and fluctuating clinical outcomes and responses to treatment, and have therefore been divided in 6 subgroups (46–48). The aggressiveness of this subtype is best exemplified by the fact that the peak risk of recurrence is between the first and third years after the initial diagnosis. Most of the deaths occur in the first 5 years following therapy (49).

Normal-like tumors are characterized by the expression of genes identical to normal mammary epithelium and shares similar immunohistochemical status with the luminal A subtype, which is ER+, PR+, HER2-, and Ki67 low. In addition, this type of tumors exhibit high expression of adipose and myoepithelium-associated genes, and low expression of luminal genes and clusters with fibroadenoma and normal breast tissue (5, 37). However, the existence of this BC subtype is controversial because the normal epithelial cell is “contaminated” with low content of malignant cells (50). In addition, the gene expression pattern of the normal breast is typified by the high expression of genes of basal epithelial and adipose cells, and the low expression of genes, characteristic of luminal epithelial cells (37).

In addition to the five BC subtypes, there is a rare and very aggressive type of BC known as inflammatory breast cancer (IBC) (51). The term inflammatory is not due to the participation of inflammatory signaling pathways (52) but for its clinical characteristics, such as the inflamed appearance of the skin. IBC is characterized by a low hormone receptor expression and is associated with a more aggressive clinical course and decreased survival (53). The aggressiveness of this disease is attributed to the alteration in key signaling pathways leading to a rapid growth and early metastasis, as well as the development of tumor emboli, which invade and block local lymphatic vessels, provoking tissue damage and immune infiltration, and also generating tumor growth (54). The clinical characteristics of IBC are diffuse induration of the skin with an erysipeloid edge, and most of the times without a dominant tumor mass (55). This behavior occurs because groups of cells are infiltrated in the stroma and they are grouped together forming clusters (56), which have characteristics of progenitor cells with CD44+ CD24−, ALDH1+, or CD133+ profiles (57).

Together, both histological and molecular systems are complementary to each other and, in combination with the TNM system, play an important role in BC diagnosis, prognosis, and therapy (23). In the next sections we will discuss the involvement of inflammatory process in the progress of BC and how this inflammation is correlated with the ROS and miRNAs production.

## Breast cancer as an inflammatory process

Inflammation is a conserved mechanism that protects from pathogens, repairs injured tissues, and eliminates damaged cells, leading to the homeostasis of the organisms (58). Acute inflammatory processes should be carefully regulated to prevent excessive tissue damage (58, 59). When acute inflammation is not resolved, it becomes chronic, causing the destruction of tissues and the disturbance of the homeostasis, leading to clinical consequences (60). Chronic inflammation is associated with the risk of developing different types of cancer, including BC (61). BC generally arise in an inflammatory environment, characterized by chronic inflammation accompanied by the presence of immune cells, proinflammatory cytokines, growth factors, and mediator proteins (8, 9).

The tumor microenvironment (TME) consists of different types of cells, including cancer cells, stromal cells, immune cells, and the extracellular matrix. The interaction among components of the TME makes it a complex system that facilitates tumorigenesis and metastasis (62). The most common immune cells found in the TME are monocytes that differentiate to tumor-associated macrophages (TAMs). TAMs have the ability to promote tumor growth and they are present during angiogenesis, invasion and metastasis; high presence of TAMs generally correlates with poor prognosis (63, 64). TAMs show strong plasticity, and they can polarize into two different populations, M1 and M2, in response to the stimuli of the microenvironment (65).

M1 macrophages are polarized by cytokines like tumor necrosis factor-alpha (TNF-α), interferon-gamma (IFN-γ), IL-1, IL-6, and IL-17, and they can produce and secrete high levels of pro-inflammatory cytokines such as IL-1α, IL-1β, IL-6, IL-12, IL-23, and cyclooxygenase-2 (COX-2). These macrophages elicit anti-tumor activity, mediate ROS-induced damage, and impair tissue regeneration and wound healing (66–68). M2 macrophages are polarized by anti-inflammatory cytokines including IL-4, IL-10, and IL-13 by the activation of signal transducer and activator of transcription 6 (STAT6) through the IL-4 receptor alpha (IL-4Rα); or by the activation of signal transducer and activator of transcription 3 (STAT3) through the IL-10 receptor (IL-10R) (69). M2 macrophages exhibit an anti-inflammatory profile, producing low levels of IL-12, high levels of IL-10, and transforming growth factor-beta (TGF-β). These macrophages have pro-angiogenic properties and potent phagocytosis capacity that promote tissue repair and wound healing (65, 69). M2 macrophages participate also in tumor progression, immunoregulation, and angiogenesis (70–72), as they secrete factors to produce an immunosuppressive TME. It is known that BC tumors possesses a high density of M2 macrophages that are associated with poor patient prognosis (73).

In addition to the M1/M2 macrophage polarization, Malyshev et al. showed that there is a third phenotype of macrophages, known as the M3 phenotype or the switching phenotype (74). They observed an imbalance in the M1/M2 alveolar macrophage phenotype, suggesting the formation of M3 phenotype. These M3 macrophages are found in lung-related conditions including bronchial asthma and chronic obstructive pulmonary disease (COPD), and during the administration of anti-inflammatory treatments like inhaled glucocorticosteroids (74–77). This M3 phenotype is characterized by the upregulation of anti-inflammatory cytokines in response to the programming factor (RF)-M1 resulting in the reprogramming to the M2 phenotype (M1/M2 phenotype). Opposite, the upregulation of pro-inflammatory cytokines in response to RF-M2 induces the reprogramming to the M1 phenotype (M2/M1 phenotype) (74). There are few reports studying the M3 macrophage population. Jackaman et al. found a mixed of IL-10^+^TNF-α^+^CD206^-^CX3CR1^+^ M1/M2 (M3) macrophage subset dominating the mesothelioma microenvironment. This observation, support the hypothesis about the transition of M1 cells to M3 cells during tumor proliferation (78). Kalish et al. reported that the M3 phenotype exhibited an antiproliferative antitumor effect *in vitro*, and prolonged the survival time of mice with Ehrlich ascites carcinoma (79). Nevertheless, more studies are needed to fully characterize and understand the activity of the M3 macrophages.

An immune cell population present also in the TME are the neutrophils. These cells constitute the first line of defense against microbial pathogens infection and tissue damage (80). In the context of TME, neutrophils are known as tumor-associated neutrophils (TANs). Depending on the stage of development of the tumor or on the tumor type, TANs could induce tumor-suppressive (N1) or tumor-promoting (N2) phenotypes (81, 82). In early stages of the tumor, neutrophils are recruited to the TME through the release of cytokines and ROS by tumor and stromal cells (83), promoting apoptosis. However, during tumor progression, neutrophils could promote angiogenesis and invasion, by modifying the extracellular matrix (ECM). TANs exerted these effects by releasing factors like proteases, reactive nitrogen species (RNS) (84), MMP9, ROS, and growth factors (i.e. VEGF) (81, 85, 86). It is reported that the chemokine receptor CXCR2 and their ligands CXCL1-3 and CXCL5-8 are in charge to attract neutrophils to the tumor and develop an inflammatory response (87). CXCR2 chemokines are produced by tumor cells, immune cells, and cancer-associated fibroblasts (CAFs) (88). It has been shown that when neutrophils are co-cultured with human BC cells, they released oncostatin M (OSM), a member of the IL-6 superfamily, promoting tumor progression, angiogenesis, and metastasis through the expression of VEGF (89). Also, the IL-17-CXCR2 axis facilitates the recruitment of neutrophils to the tumor site, promoting BC progression (90).

Another cells found in the TME are myeloid-derived suppressor cells (MDSCs), a heterogeneous group of monocytic and polymorphonuclear immature myeloid cells that are produced under inflammation (91). MDSCs participate in premetastatic niche formation and promote tumor cell metastasis and angiogenesis by secreting TGF-β, VEGF, and MMP9 (92, 93). MDSCs can also inhibit immune function to accelerate tumor progression (92, 93), increase tumor cell stemness and angiogenesis, as well as promoting EMT through IL-6 secretion (94). In cancer patients, the expansion of MDSCs promotes cell growth and metastasis, and decrease the immunotherapy effectiveness (91). It has been reported that high levels of MDSCs in BC correlates with advanced stages of the disease and increased rates of recurrence and metastasis (95). It has been observed that in the lung tissues of BC patients, CCL2 can recruit MDSCs to promote BC metastasis (96).

T cells are also found in the TME, and they are classified into CD8+ cytotoxic T cells and CD4+ helper T cells, including Th1, Th2, Th17, T regulatory cells, as well as natural killer T cells. Importantly, T cells have tumor-suppressive and tumor-promoting capabilities (97, 98). In addition, the γδ T cells are also found in the TME, they are known as nonconventional lymphocytes and characterized by the expression of specific receptor of the Vγ and Vδ chains (99). The presence of γδ T cells in the TME is associated with poor prognosis in different types of cancer, including BC. Chabab et al. observed that in human BC tumors, γδ T cells were present in the late stages of the disease (100). Around ~20% of the γδ T cells expressed CD37, and exhibited immunosuppressive functions through the expression of IL-8, IL-10, and adenosine (100).

CAFs are also part of the BC stromal compartment and are recruited and activated by different mediators, including TGF-β, platelet-derived growth factor (PDGF), fibroblast growth factor 2 (FGF-2), and ROS (101). CAFs are able to secrete proteases, inflammatory molecules and growth factors. These proteases and inflammatory molecules increase the ability of CAFs to migrate and remodel the ECM, and recruit other inflammatory cells at neoplastic sites (102, 103). The growth factors also stimulate cancer cells to produce and secrete TFG-β, MMP9 and MMP13 which contribute to the proliferation and angiogenesis of the tumor (104). In BC, high levels of MMP9 are associated with cancer development and tumor progression, while in TNBC MMP9 promotes angiogenesis and metastasis (105, 106).

The TME also includes cancer-associated adipocytes (CAAs). CAAs are smaller than normal adipocytes, and also differs in metabolic activity and adipokine expression (107). CAAs secrete adipokines, inflammatory chemokines, and interleukins and are implicated in tumor progression, metastasis, and therapy resistance (108). For instance, it has been shown that high secretion of leptin by CAAs promote cell proliferation and angiogenesis in BC by the upregulation of the enzyme lysyl hydroxylase (109). Leptin in CAAs also activates ER pathways, JAK/STAT3, PI3K/AKT and SRC-1 signaling pathways and increases cyclin D1 and vascular endothelial growth factor (VEGF)/VEGFR expression (108, 109). Increased levels of STAT3 and JAK phosphorylation in CAAs may led to the production of IL-6, which promotes cancer cell survival, immune suppression, and drug resistance (110).

Mediators of the immune response and inflammatory mediators such as ROS, cytokines, chemokines, prostaglandins, and growth factors can regulate cancer progression (111), and participate in all the stages of carcinogenesis, including initiation, promotion, cell proliferation, angiogenesis, and metastasis (11, 112, 113). TGF-β is a cytokine that also promotes metastasis in BC and it is crucial during epithelial-mesenchymal transition (EMT), invasion, and progression of BC (114). TNF-α is known as a multifunctional cytokine that contributes to cancer development and also participates in different signaling pathways connected to inflammation, proliferation, survival, invasion, and migration in BC (115). The effects of TNF-α are mediated through its receptors, TNFR1 and TNFR2 (116). The binding of TNF-α to its receptors activates the p42/p48 MAPK, PI3K/AKT and p38/MAPK pathways which activate the expression of proliferation-related genes mediated by NF-kB, STAT3, and AP-1 transcription factors (117). Resistin, another inflammatory cytokine, is released in the inflammatory TME, promote tumor cell growth and aggressiveness, and it is elevated in BC patients (118, 119). High levels of resistin positively correlated with breast tumor size and stage, lymph node metastasis, and estrogen receptor status, and negatively correlated with the overall survival in BC patients (120).

Some ILs as IL-1, IL-6, IL-8, IL-11, and IL-23 also promote an inflammatory microenvironment, and some of them are involved in tumor progression (121). IL-6, a pleiotropic cytokine that regulates multiple biological activities is the most studied cytokine in the pathogenesis of BC (122). It is speculated that IL-6 can inhibit apoptosis by regulating the expression of antiapoptotic proteins, such as B-cell lymphoma-extra-large (BCL-XL) and B-cell lymphoma 2 (BCL-2) (123). IL-6 also promotes cell survival, immune suppression, and drug resistance through STAT3 dependent pathway and JAK phosphorylation (123, 124). The JAK/STAT3 signaling pathway is induced by the overexpression of IL-6 released from TAMs, causing the translocation of phospho-STAT3 into the nucleus and transactivation of proteins, inducing the processes of proliferation, differentiation, and survival (125, 126). Another cytokine involved in inflammation and cancer progression is IL-17. This cytokine protects the body against infections. IL-17 promotes inflammation by inducing inflammatory mediators such as IL-6, CXCL1 and G-CSF (127), recruiting dysfunctional myeloid cells and establishing a suppressive and proangiogenic immune response in the TME (128). Dysregulation of IL-17 production or in its signaling pathway causes an unresolved inflammation, resulting in tissue damage. The chronic activation of IL-17 generates a pro tumor microenvironment, since IL-17 induces inflammatory mediators to promote tumor progression (128).

Other mediators of inflammation in the TME are the chemokines and their receptors. These molecules are implicated in diverse processes, including wound healing, angiogenesis, inflammatory diseases, tumor growth, and metastasis (129, 130). The chemokine CCL20 and its receptor CCR6 promotes cancer progression by increasing the proliferation and migration of cancer cells (131). CCL20 has been associated with poor prognosis in BC patients, Lee et al. reported that patients with high expression of CCL20 showed significantly lower overall survival and metastasis-free survival, in addition to increase cell invasion and secretion of MMP2 and MMP9 in TNBC cells (132). The chemokine CXCL8 and their chemokine receptors CXCR1/2 are also fundamental in the activation and trafficking of inflammatory mediators, and tumor progression as well as metastasis (133). It has been reported that in BC the chemokine CXCL8 induces EMT (134), and participates in angiogenesis, cell invasion, and migration of TNBC cells (135). The chemokine receptors CXCR2, CXCR3, CXCR4, CCR6, and CCR7 and their cognate ligands are associated to BC metastasis (130, 136, 137).

In addition to cytokines and chemokines, lipid mediators, derived from polyunsaturated fatty acids (i.e. arachidonic acid, eicosapentaenoic acid, and docosahexaenoic acid) that are synthesized during normal cell homeostasis, they are also over-produced under stress conditions, and contribute to the inflammation and tumor progression (138, 139). Prostaglandins are derived from arachidonic acid and are catalyzed by cyclooxygenases (COX) (139). Evidence indicates that prostaglandin E_2_ (PGE_2_), the most abundant prostaglandin, participates in several carcinogenesis-related processes including cell growth, apoptosis escape, EMT, and angiogenesis (140, 141). PGE_2_ is able to bind to four G-protein-coupled EP receptors, EP1-EP4. EP4 is frequently upregulated in cancer cells where it promotes proliferation, migration, invasion, and metastasis (142). In murine pulmonary endothelial cells, PGE_2_ enhanced tumor metastasis by promoting release of VEGF through the EP2 receptor pathway (143). EP4 stimulation increased proliferation, invasion, and metastasis of SUM149 IBC tumor cells, and was correlated with aggressive BC subtypes (144, 145).

Besides the role in TME, inflammation also promotes the overproduction of ROS which contribute to BC progression. The interactions between inflammation and ROS in the context of BC is discuss in the next section.

## Interplay between inflammation and ROS production in breast cancer

ROS are free radicals, ions or molecules with a single unpaired electron (12); these oxygen-containing molecules are small, short-lived and highly reactive (146). There are more than 20 types of ROS classified into two groups, free oxygen radicals and non-radical ROS (147, 148). The most studied ROS associated with cancer are superoxide anions and hydroxyl radicals of the free oxygen radical group, and hydrogen peroxide of the non-radical ROS group (146).

Intracellular ROS are mainly originated by the mitochondria’s electron transport chain. The endoplasmic reticulum, lysosomes, and peroxisomes also produce considerable amounts of ROS (149). ROS have a dual role; at low levels, ROS are signaling molecules in different physiological events including cell growth and survival, apoptosis, and immune response (13, 14). When ROS levels are elevated, for example during a chronic inflammatory event, they induce damage to the DNA, proteins, and lipids. DNA damage results in genetic instability, tumorigenesis and aging (147).

During an inflammatory process associated with the initiation and progression of BC, macrophages and mast cells of the TME produce inflammatory mediators that increase vascular permeability, allowing the migration of leukocytes to the site of damage (150). ROS participate in this process by regulating the expression of molecules such as intercellular adhesion molecule 1 (ICAM-1), vascular cell adhesion molecule 1 (VCAM-1), P- and E-selectin that are expressed in the endothelial surface and interact with leukocytes, favoring their migration (151). Other molecules that induce cell migration and adhesion are cytokines such as TNF, platelet-derived growth factor, angiopoietin-1, and VEGF. The binding of these chemoattractants and growth factors to cell surface receptors triggers nicotinamide adenine dinucleotide phosphate (NADPH) oxidases to form ROS (152, 153). Additionally, ROS can induce the activation and synthesis of factors responsible of the inflammatory response including hypoxia-inducible factor-1 alpha (HIF-1α), β-catenin/Wnt, activator protein 1, NF-kB, peroxisome proliferator-activated receptor gamma, growth factors, and pro-inflammatory cytokines (154–157).

ROS overproduction plays also important role in EMT. During EMT, epithelial cells lose their junctions and their polarity, a cytoskeleton reorganization occurs, and a reprograming of gene expression that promote mesenchymal properties including cell motility and invasive phenotypes (158, 159). The TGF-β signaling pathway play an important role in EMT and higher levels of TGF-β1 and TGF-β receptor type II (TβRII) have been observed in BC cells. The pathway through which TGF-β drives EMT and cellular migration in breast epithelial normal and tumor cells partially rely on the NADPH oxidase 4 (NOX4) (160, 161), an enzyme, that is a major source of intracellular ROS (162). Zhang et al. reported that in the BC cells 4T1, TGF-β induced ROS production and enhanced cell migration (160). These TGF-β-associated effects were mediated by NOX4 because an NOX4 chemical inhibitor and RNAi-mediated NOX4 knockdown significantly decreased TGF-β-dependent cell migration (160). Tobar et al. co-cultured RMF-EG mammary stromal cells and MCF-7 cells to observe the migratory ability of the breast cancer cells. Pre-treatment of RMF-EG cells with TGF-β1 enhanced the migratory ability by elevating NOX4 expression and intracellular ROS production. These effects were abolished by knocking down NOX4 in RMF-EG cells with small interfering RNA (siRNA) (163). Other reports indicate that TGF-β1 regulates uPA (Urokinase type Plasminogen Activator) and matrix metalloproteinase 9 (MMP-9) using ROS-dependent mechanisms (164, 165). Additionally, in MCF-7 cells ROS increased tumor migration by inducing MMPs-mediated hypoxia and cathepsin expression (166, 167).

In general, the high levels of ROS, generated by increased mitochondrial metabolic and energetic activity, by alterations in the electron transport chain, by HIF-1α expression, and/or during a chronic inflammation (168), are responsible for increasing the activation of the PI3K/AKT and MAPK/ERK signaling pathway and cell proliferation (147). Incubation of MDA-MB-231 BC cells with deferoxamine (DFO) increased ROS levels, activated the ERK signaling pathway, increased HIF-1α expression, and promoted cell migration and invasion of these cells (169). A study by Han et al. found that high levels of epidermal growth factor (EGF) promoted the production of hydrogen peroxide (H_2_O_2_), and activated p70S6K1 *via* the PI3K/AKT. This signaling pathway promoted also the VEGF and HIF-1α production in MCF-7 cells (170). In another report, copper, which in excess is a potent oxidant causing the production of ROS in cells (171, 172), through the EGFR/ERK/c-Fos pathway, increased the expression of VEGF, HIF-1α, and G-protein estrogen receptor (GPER) in the SKBR3 BC cells (173, 174). Also, in MCF-7 cells ROS activated the PI3K/AKT signaling pathway and increased the expression of HIF-1α and angiogenesis (175). Together, ROS stimulate growth factors, cytokines, and molecules such as HIF-1α and VEGF, which induce migration and proliferation of BC cells (176–178). These ROS-dependent signaling pathways activate the PI3K/AKT/mTOR pathway, increasing VEGF production by HIF-1α dependent and independent mechanisms.

Opposite, ROS are able to induce apoptosis by destabilizing the mitochondrial membrane and opening the mitochondrial permeability transition pore. These events alter the electron transport chain and releases cytochrome-c that, in conjunction with apoptotic peptidase activating factor 1 and procaspase-9 forms apoptosomes. Apoptosomes activate caspase-9, and then caspase-3 which executes the last steps of the apoptosis cascade in the nucleus (179–182). As apoptosis is a desirable mechanism to eliminate undesirable cells, the generation of ROS could be beneficial to eradicate cancer cells in tumors (183–185). Some anticancer agents, such as the natural polyphenol resveratrol, promotes apoptosis of MCF-7 cells through the accumulation of H_2_O_2_ in the mitochondria (186).

Together, ROS have a central role in the growth and proliferation of cancerous cells as well as in the TME as mediators of the oxidative stress conditions, and in the inflammatory responses. ROS are not the only molecules involved in these meaningful processes as miRNAs are also able to regulate ROS production. In the next section, we will discuss the interconnection between miRNAs in ROS production during BC progression.

## Interplay between miRNAs and ROS in breast cancer

### Deregulation of miRNAs in breast cancer

Although only 2% of the human genome is composed of protein coding genes, more than 90% of the DNA is transcribed into RNA (187). RNA molecules that are not translated into proteins are called non-coding RNAs (ncRNAs), whose biological and regulatory functions are still under investigation (188, 189).The first report that ncRNA participate in the regulation of gene expression occurred in 1984 by Mizuno et al. who observed that the *Escherichia coli micF* gene has its own promoter and encodes a small ncRNA that can inhibit translation of *ompF* mRNA by base pairing (190, 191). In the subsequent years it became evident that ncRNAs are a key piece in the regulation of gene expression.

Depending on their function, ncRNAs are classified into two major groups (192).The first group are the housekeeping ncRNAs, which regulate essential cellular functions and includes ribosomal RNA (rRNA), transfer RNA (tRNA), small nuclear RNA (snRNA), small nucleolar RNA (snorRNA), and telomerase RNA (TERC) (193). The second group are the regulatory ncRNAs, which regulate gene expression at virtually every level (194). Depending on their size, regulatory ncRNAs are subclassified into small ncRNAs (sncRNAs) which are less than 200 nt in length, and long ncRNAs (lncRNAs) with more than 200 nt of length (193). Regulatory sncRNAs include, tRNA-Derived Fragments (tRE), halves tRNA (tiRNA), siRNA, piwi-interacting RNA (piRNA), enhancer RNA (eRNA), circular RNA (circRNA), Y RNA, and miRNAs. MiRNAs are the most studied sncRNAs due their critical role in the regulation of gene expression (188).

MiRNAs are small non-coding RNAs of about 22 nucleotides in length that regulate gene expression at the posttranscriptional level (18). Most miRNAs are transcribed by RNA polymerase II/III to generate a primary transcript (pri-miRNA) which is processed by microprocessor complex formed by Drosha-DiGeorge syndrome critical region gene 8 (DGCR8) to produce an RNA stem-loop pre-miRNA of ~80 nucleotides in length. Pre-miRNAs are transported into the cytoplasm by RanGTP/exportin 5 (XPO5), where Dicer together with the transactivation response RNA binding protein (TRBP) generate a mature miRNA duplex. The duplex is loaded into Argonaute (AGO) proteins to form the RNA-induced silencing complex (RISC). RISC promotes miRNA molecules to bind their target mRNA. The ability to regulate gene expression depends on their degree of complementarity between the miRNA and the 3’UTR region of the mRNA target. A total complementary between mRNA and miRNA lets to RNA degradation but, incomplete complementarity causes a partial repression of translation (19).

Deregulation of miRNAs is a phenomenon observed in most cancers, including BC, and has been linked with all steps of carcinogenesis and drug resistance (20). Many deregulated miRNAs have been proposed as diagnostic or prognostic markers and/or as targets for BC therapy (20). In cancerous cells, upregulated miRNAs are known as oncomiRs and generally reduce the expression of genes with tumor suppressor capabilities. Opposite, downregulated miRNAs are known as tumor suppressor miRNAs and due to their absence, their target genes are upregulate acting as oncogenes (19). For instance, miR-145 promotes TNF-α-induced apoptosis in triple negative BC by facilitating the formation of RIP1-FADD/caspase-8 complex (195). On the other hand, miR-146b inhibits apoptosis in BC cells by inhibiting STAT3 and reducing IL-6 production in a NF-kB dependent manner (196). Moreover, TGF-β can cause an increase in the expression of miR-106b, which promotes tumor growth and metastasis in BC (197).

As it was above mentioned, inflammation plays a fundamental role in BC progression (198). However, the role of miRNAs in this process is still not fully understood. A decade ago, O’Neill et al. described that immune cell populations expressed miRNAs that act on target genes involved in the regulation of the inflammatory process. Such miRNAs acts on mRNA of inflammatory-related-molecules, including: (i) receptors such as Toll-like receptor 4 (TLR4) (miR-223, let-7i, let-7e), TLR3 (miR-223), TLR2 (miR-105), (ii) signaling molecules such as myeloid differentiation primary-response protein 88 (miR-155), MYD88 adaptor-like protein (miR-145), IL-1R-associated kinase 1 and 2 (miR-146), TNFR-associated factor 6 (miR-146), (iii) transcription factors such as nuclear factor-κB1 (miR-9), forkhead box P3 (miR-155), p300 (miR-132), (iv) cytokines such as IL-6 (let-7), TNF (miR-16, miR-155, miR-125b, miR-579, miR-369-3), IL-10 (miR-106, miR-466l), IL-12p35 (miR-21), and (v) regulator proteins such as acetylcholinesterase (miR-132), programmed cell death 4 (miR-21), Src homology 2 (SH2) domain-containing inositol-5ʹ-phosphatase 1 (miR-155), and suppressor of cytokine signaling 1 (miR-155) (199, 200). Later, it was recognized that there is a feedback between miRNAs and the immune system to regulate the inflammatory responses and to protect the host from inflammation (201).

Depending on the type and differentiation stage of cells and tissues, a particular miRNA is expressed at different magnitudes. Also, a miRNA, could be regulated through different mechanisms in normal *vs*. malignant cells. The innate immune system has the ability to regulate the expression of multiple miRNAs (202). During an inflammatory response, various proinflammatory, signaling pathways, and transcription factors are activated. For example, activation of NF-kB in macrophages, DCs, and THP-1 cells, upregulates miR-21, miR-146a, and miR-155 (203–205). Enzymes related to inflammatory processes including adenosine deaminase RNA Specific (ADAR1) and Polyribonucleotide Nucleotidyltransferase 1 (PNPT1) are involved in the processing of pri-miRNAs (202).

In some populations of the innate immune system, miRNAs play important roles. Diverse technologies have been used for the analysis of miRNAs and their relationship with the immune system. For instance, Ishii et al. used a NanoString miRNA array to measure the miRNA expression levels in Gr-1+CD11b+ human myeloid cells and found that miR-130a and miR-145 can reprogram tumor-associated myeloid cells by modifying the cytokine milieu and the metastatic microenvironment. These two miRNAs regulate TGF-β receptor II production that results in an enhanced antitumor immune response (206). Chiodoni et al. used Bead array technology (Illumina), to assess whether circulating miRNAs from bone marrow samples were involved in the communication between the nascent cancer and the bone marrow. They identified 80 miRNAs differentially expressed between NeuT mice and the control group. Some of these miRNAs could be responsible for transcriptional alterations in the bone marrow that favor an environment of immunosuppression during cancer development. These results provide evidence that deregulated miRNAs alters the immune system in response to the carcinogenesis process (207). Qing et al. found that Kaposi’s sarcoma-associated herpesvirus encodes two miRNAs: miR-K12-3 and miR-K12-7. These two miRNAs bind to the 3´UTR of the basic region/leucine zipper motif transcription factor C/EBP and regulate its expression. Upregulation of C/EBP increased IL-6 and IL-10 expression in infected macrophages which resulted in suppression of antitumor immune responses, promotion of tumor cell growth, suppression the T cells activation, and increased angiogenesis (208).

Another effect of miRNAs on the immune response is their regulatory effect on IL-17. Seif et al. found that overexpression of miR-490-5p and miR-490-3p in peripheral blood mononuclear cells (PBMC) and plasma from BC patients led to the production of Th17 lymphocytes and IL-17-producing regulatory T cells (Tregs). This immune response reduced the levels of *CD3d, IL-2, IL-2 receptor chain alpha, forkhead box O1 (FOXO1)*, and *nuclear factor of activated T cells 5 (NFAT5)*; a microenvironment that supports tumor progression (209). Soheilifar et al. reported that overexpression of miR-182-5p and miR-182-3p suppresses the FOXO1, NFATs, and IL-2/IL-2RA signaling pathways. On the other hand, these two miRNAs increased the expression of FOXP3, TGF-β, and IL-17 in tumor tissues engender IL-17-producing Tregs that exert immunosuppressive functions in BC (210).

An effect of miRNAs on some inflammatory mediators such as PGE2 has been reported (211). Kim et al. found that miR-155 is involved in the metabolism of prostaglandins, as this miRNA up-regulates PGE2-producing enzymes (PTGE S/PTGES2) and down-regulates PGD2-producing enzymes (PTGDS). The correlation between high levels of miR-155 and overexpression of PGE2/prostaglandin D2 (PGD2), confirm the importance of this miRNA in the regulation of inflammation during carcinogenesis (211). COX-2 overexpression has been also related to the expression of miRNAs such as miR-526b and miR-655 in an MCF-7-COX2 BC cell line (212, 213). Overexpression of both miRNAs induced stem-like cell phenotypes, and stimulated angiogenesis (212, 214). Furthermore, Hunter et al. reported that the overexpression of miR-526b and miR-655 promoted the expression of angiogenic markers such as VEGF and EP4 receptors (215, 216).

As mentioned above, multiple cytokines and chemokines (TNF-α, TGF-β, granulocyte-macrophage colony-stimulating factor (GM-CSF)), as well as transcription factors (AP-1, NF-kB, STAT3, HIF-1α), participate in inflammatory responses during cancer progression. MiRNAs posttranscriptionally regulate the expression of those molecules. For example, miR-421 (targets: *FXR, DPC4/SMAD4, ATM*), miR-503 (target: *CCND1*), miR-24-3p (targets: *p27, p16*), miR-29a (targets: *PTEN, GSK3β, TET1*) and miR-451 (targets: *MIF, YWHAZ*) play a critical role in the proliferation, migration, invasion, and metastasis of BC. Additionally, miRNAs have a direct effect on ROS regulation, as will be seen in the next section (217–219).

### MiRNAs alter ROS production in breast cancer

Deregulation of miRNAs contributes to the production of ROS and therefore to BC progression. For example, Moi et al. reported that the nuclear transcription factor erythroid-derived factor 2-like 2 (Nrf2) and its Kelch-like inhibitor ECH-associated protein 1 (Keap1) (220) are regulators of the oxidative stress responses (221, 222). Under basal conditions of oxidative stress, Nrf2 is sequestered by Keap1 and directed to the proteasome (223). However, if ROS levels increase the Nrf2/Keap1 complex separate and Nrf2 is transferred to the nucleus, where it promotes the expression of encoding antioxidative-related genes such as proteins involved in redox balancing factors, detoxifying enzymes, stress response proteins and metabolic enzymes (223). This mechanism keeps cancer cells from entering apoptosis and favors tumorigenesis (224, 225). Interestingly, Singh et al. reported that Nrf2 is downregulated by miR-93 (226). The overexpression of miR-93 in breast epithelial cells decreases apoptosis and increased colony formation, mammosphere formation, cell migration, and DNA damage. Opposite effects were observed after miR-93 reduction with vitamin C, an agent that reduce the intracellular levels of ROS (226). Yang et al. found a negative relationship between the expression levels of miR-28 and Nrf2. MiR-28 regulate the expression of Nrf2 in a Keap1- independent manner (227). Eades et al. reported that miR-200a binds to *KEAP1* mRNA, which increase the expression of Nrf2 in BC (228). Yi et al. reported that miR-101 inhibits Nrf2 expression and suppressed the proliferation of BC cells, making them more sensitive to oxidative stress (229). Overexpression of miR-153 reduced Nrf2, inhibited apoptosis and increased colony formation in BC cells (230). The **Table 2** summarizes the reported miRNAs that regulate genes involved in the redox signaling pathway in BC (**Table 2**) (228).

**Table 2:** miRNAs involved in ROS regulation in Breast Cancer.

Pathway	MiRNA	Action	Effect	Reference
**Nrf2/Keap1**	miR-93	Nrf2 downregulation	Decreases apoptosis, promotes colony and mammosphere formation, increases cell migration and DNA damage	(226)
	miR-28	Nrf2 downregulation	Increases colony formation	(227)
	miR-200a	Keap 1 downregulation		(228)
	miR-101	Nrf2 downregulation	Suppresses cell proliferation	(229)
	miR-153	Nrf2 downregulation	Decreases apoptosis and increases colony formation	(230)
**NF-kB**	miR-520/373	NF-kB, TGF-β	Tumor suppression	(231)
	miR-31	PKCϵ	Sensitizes to apoptosis	(232)
	miR-30c-2-3p	TNFR/NF-kB	Reduce proliferación e invasion	(233)
	miR-1246	PKA/PP2A	NF-kB pro-inflammatory signaling	(234)
	miR-221/222	PTEN	Promote stem-like properties and tumor growth	(235)
***M*itochondria and metabolism**	miR-195	Acts on *ACACA, FASN*, *HMGCR, CYP27B1*	Decreases proliferation, invasion, and migration	(236)
	miR-485-3p miR-485-5p	Act on peroxisome proliferator-activated receptor-gamma Coactivator-1 alpha (PGC-1α)	Inhibits migration, invasion, and metastasis	(237)
	miR-27b	Suppress PDHX	Promotes cell proliferation	(238)
	miR-3677	TLE3 downregulation	Induces proliferation, migration, and metastasis	(239)
	miR-4485	Acts on 16S Rrna	ROS accumulation	(240)
	miR-342-3p	MCT1	Disrupt energetic fluxes	(241)
	miR-155/miR-143	C/EBPβ	HKII expression	(242)
	miR-204-5p	PIK3CB	Regulates growth, metastasis, and immune microenvironment remodeling	(243)
**Hypoxia**	miR-153	HIF-1α	Inhibits migration, proliferation, and angiogenesis	(244)
	miR-191	TGF-β	More aggressive tumor	(245)
	miR-18a	HIF-1α	Reduces metastasis	(246)
	miR-497	HIF-1α, VEGF	Reduces tumor growth and angiogenesis	(247)
**Other**	miR-509	Inhibits SOD2	Inhibits proliferation, migration, and angiogenesis	(248)
	miR-500a-5p	TXNRD1 and NFE2L downregulation	Promotes the progression of BC	(249)
	miR-139-5p	Decreases MAT2A	Synergy with radiotherapy, increases ROS production	(250)
	miR-526b miR-655	*TCF21* and *PBRM1*	TXNRD1 upregulation	(251)

MiRNAs also regulate diverse mitochondrial genes that encode proteins involved in cellular redox homeostasis (see **Table 2**). Singh et al. overexpressed miR-195 in MCF-7 and MDA-MB-231 BC and performed a gene expression profile. They observed mitochondrial dysfunction due to alterations in fatty acid metabolism or xenobiotic metabolism (236). There was also a reduced expression of various miR-195 target genes including *ACACA*, *FASN*, *HMGCR*, and *CYP27B1* (236). Additionally, they observed a reduction in cell proliferation, invasion, and migration, a reduction in mesenchymal markers, and an increasing in epithelial markers (236). Lou et al. found that both miR-485-3p and miR-485-5p act on peroxisome proliferator-activated receptor-gamma coactivator-1 alpha (PGC-1α), a key element in regulation of cellular energy metabolism (237). The absence of this miRNA increased BC migration, invasion, and metastasis (237). Eastlack et al. found that increasing the miR-27b expression inhibited pyruvate dehydrogenase protein X (PDHX) in BC, which leads to altered levels of pyruvate, lactate, and citrate. Increased levels of miR-27a also reduced mitochondrial oxidation and promoted extracellular acidification and cell proliferation (238). More recently, Peng et al. reported that the overexpression of miR-3677 induced proliferation, migration, and metastasis in BC. In this setting, miR-3677 downregulates the expression of transducin-like enhancer of Split3 (TLE3), a transcriptional co-repressor involved in cell proliferation, metabolism regulation, and tumorigenesis (239). This observation suggest that miR-3677 acts as oncomiR in BC by increasing ROS levels (239). On the other hand, during oxidative stress conditions, miR-4485 could be translocated into the mitochondria where it binds to 16S rRNA, leading to an accumulation of ROS and mitochondrial dysfunction. In the mitochondria, miR-4485 regulated the activity of mitochondrial complex I, altered ATP production, activated caspase-3/7, and induced apoptosis. Because miR-4485 is downregulated in BC, its overexpression has been associated with suppression of tumorigenesis (240).

Many other miRNAs can alter the oxidative stress conditions inside cells. Song et al. found that miR-509 overexpression inhibits proliferation, migration, and angiogenesis in BC cells by downregulating superoxide dismutase 2 (SOD2), a central protein involved in ROS production (248). MiR-500a-5p overexpressed under conditions of oxidative stress, downregulates thioredoxin reductase 1 (TXNRD1) and nuclear factor erythroid 2-like 2 (NFE2L2), two proteins involved in stress responses, whose reduction is related to poor prognosis in BC (249). Transfection of MCF-7 cells with a miR-139-5p mimic followed by radiation increased ROS production and induced apoptosis in BC due to its action on methionine adenosyltransferase 2A (MAT2A). On the other hand, mice treated with a miR-139-5p mimic, completely eliminated the implanted tumor and they remained tumor-free (250).

Shin et al. found that overexpression of miR-526b and miR-655 in MCF-7 cells increased the expression of TXNRD1, and promoted ROS production, for this reason, it could be involved in the tumor growth and metastasis in BC (251). MiR-526b targets *TCF21* whereas miR-655 targets *PBRM1*. Both, *TCF21* and *PBRM1* are negative regulators of TXNRD1, since they inhibit the TXNRD1 expression (**Table 2**). Furthermore, MCF-7 cells treatment with H_2_O_2_ promoted the overexpression of these two miRNAs, ratifying that oxidative stress are able to induce the expression of miRNAs (251).

**Figure 1:**
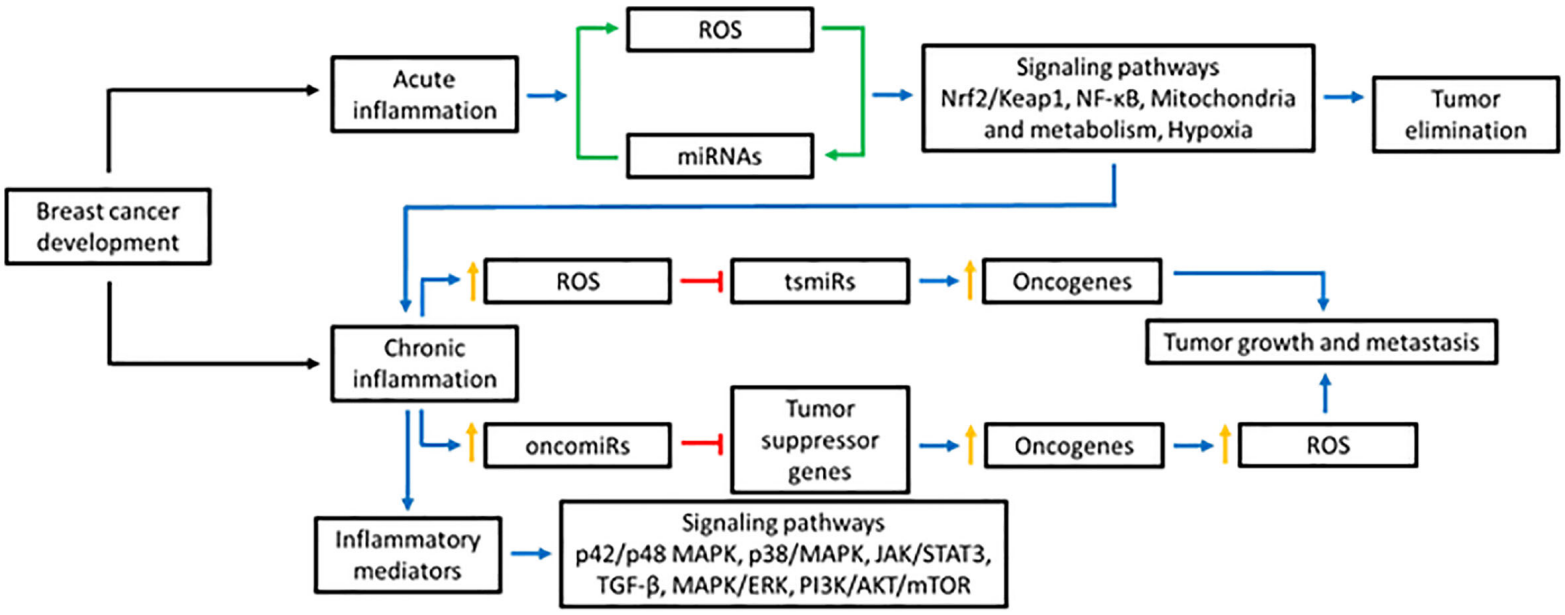
Effect of ROS in the miRNAs production. ROS can affect the production of miRNAs in various ways: modifying the miRNA biogenesis through the action on DGCR8 and DICER, altering the expression of transcription factors responsible for regulating their expression, or causing epigenetic alterations. Blue lines represent activation, red lines inhibition, and green lines bidirectional regulation.

Together, miRNAs are important regulators of ROS and BC progression. The close relationship between miRNAs and ROS is essential to maintain cellular homeostasis. Alterations in miRNA and/or ROS levels could promote BC progression (**Figure 1**).

### ROS alter miRNAs in breast cancer

**Figure 2:**
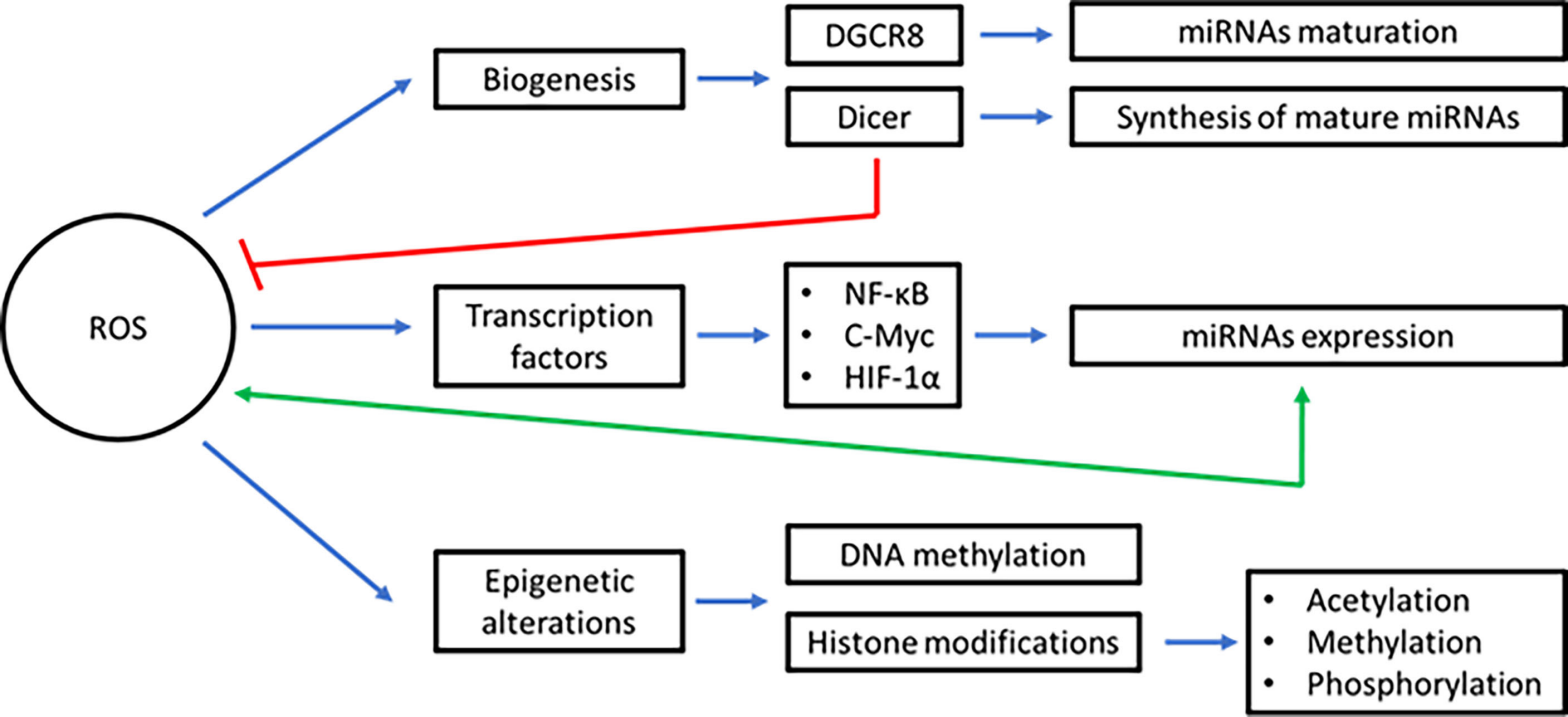
Interplay between inflammation, ROS, and miRNAs in BC. To prevent the development/progression of BC, acute inflammation must be resolved. ROS and miRNAs play a key role in this inflammatory mechanism. Instead, during tumor development and tumor maintenance, chronic inflammation occurs. In this process, several inflammatory mediators are generated that deregulate ROS and miRNAs. Furthermore, the production of ROS and miRNAs are interconnected each other and with the inflammatory mediators during all steps of the carcinogenesis process in BC. Blue lines represent activation, red lines inhibition, green lines bidirectional regulation, and orange lines overexpression. (oncomiRs: oncogenic miRNAs, tsmiRs: tumor suppressor miRNAs).

Excess of ROS production alters the levels and function of virtually all biomolecules in cells, including miRNAs (22). Early evidence showed alterations in miRNA expression in response to radiation or H_2_O_2_ in cultured normal human fibroblasts (252). This effect was prevented using cysteine, which acts as an scavenger of free radicals (252). Oxidative stress can induce or decrease the expression of miRNAs through different mechanisms including the alteration of the biogenesis process, deregulation of transcription factors, or inducing epigenetic alterations (253) (**Figure 2**). For instance, ROS acts on heme groups linked to the heme-binding motif of DGCR8, causing a conformational change that suppresses pri-miRNA processing activity (254–256). Another protein downregulated by ROS is Dicer, which is overexpressed by the action of Nrf2 and had a direct effect on the synthesis of mature miRNAs (257). Let-7, a miRNA overexpressed under oxidative stress conditions, has the ability to suppress the action of Dicer, which is one of its target genes (258). Moreover, it has been reported that aging and ROS alter Dicer expression in cerebromicrovascular endothelial cells. Exposition of these cells to H_2_O_2_ reduced Dicer expression and downregulate miRNAs synthesis in around 89% when analyzing the miRNAs expression levels by RT-qPCR (259). Dicer could also works as a negative regulator of ROS production, a mechanism used to maintain redox balance (260). On the other hand, human microvascular endothelial cells with Dicer knockdown exhibited increased levels of HMG-Box Transcription Factor 1 (HBP1). HBP1 acts on p47phox, a subunit of NADPH oxidase, causing a decrease in ROS production and affecting angiogenesis in endothelial cells (260).

Interestingly, ROS are also capable of modifying mature-miRNAs. For example, under oxidative stress conditions, miR-184 suffers an oxidation process that allows it to interact with the 3’UTR region of anti-apoptotic proteins such as BCL-XL and B-cell lymphoma-w (BCL-W). Under normal conditions, genes encoding these proteins are not targets of miR-184 (261).

The expression of miRNAs is regulated by transcription factors such as NF-kB, cellular myelocytomatosis oncogene (c-Myc), AP-1, and HIF-1α, among others. The expression of many of these transcription factors is at the same time altered under oxidative stress conditions (254). For instance, reduced levels of miR-148a and miR-152 caused an increase in the expression of NF-kB (262). By a feedback loop, increased levels of NF-kB increased the levels of those miRNAs which could promote the growth and angiogenesis in BC (262). A CuO nanowire fabricated with folic acid (CuO-Nw-FA) caused a reduction in the NF-kB levels that reduced the expression of miR-425 and caused the overexpression of *PTEN*, a miR-425 target gene (263).

Another oxidative stress-related protein involved in the regulation of miRNAs expression is the endogenous protein kallistatin. Kallistatin promotes the expression of genes with antioxidant activity by at least two reported ways (264). First, kallistatin reduces the expression of TGF-β, through its active site and promotes the synthesis of endothelial nitric oxide synthase (eNOS), reducing oxidative stress, a powerful inducer of EMT process (264). Equally, TGF-β decreases the levels of miR-21, a well-known oncomiRNA, that targets PTEN, promoting chemoresistance and invasion (264, 265). Second, kallistatin through its active site stimulated the synthesis of eNOS/Sirt1/FoxO1, a pathway essential to regulate the oxidative stress conditions inside cells (264). Chao et al. found that kallistatin also stimulates the expression of miR-34a and *p53*, both inhibits the miR-21/AKT/BCL-2 pathway, leading to apoptosis in BC cells (266).

In addition to the effect of ROS on miRNA biogenesis, miRNAs are regulated by ROS at the epigenetic level (254). Some of the epigenetic alterations contributing to the miRNA dysregulation in cancer include DNA methylation and histone modifications (acetylation, methylation, and phosphorylation) (267). DNA methyltransferases (DNMTs) and histone deacetylases (HDACS) are two of the major enzymes epigenetically modifying DNA and histones, respectively (254). Han et al. measured the expression of miRNAs in cell lines that were knockout for DNMT1 and DNMT2 and found that the expression of about 10% of miRNAs are dependent on the DNA methylation status (268). Methylation of CpG regions is also important in the regulation of the expression of miRNAs, where the participation of DNMT3A and DNMT3B has been seen, by not allowing the expression of some regions of the genome (269). Oxidative stress may lead to HDACs degradation, causing changes in the miRNAs expression pattern (270, 271). Oxidative stress caused by glucose deprivation in mouse cells (B/CMBA.Ov) inhibited HDAC2 and induced the acetylation of the promoter region of miR-466h-5p and increasing its expression. These cells exhibited apoptotic features, because miR-466h-5p reduced the expression of some target genes with anti-apoptotic activity such as *bcl212*, *dad1*, *smo*, *birc6* and *stat5a* (270, 272).

To summarize, there is an intricate interconnection between miRNAs and ROS as together they form complex bidirectional regulatory pathways that must be properly regulated to maintain cell homeostasis. Alterations in these pathways can lead to the progression of BC.

## Opportunities for therapy

Due to the multiple feedback loops between ROS, miRNAs, and inflammation in the progression and tumor maintenance of BC cells, both ROS and miRNAs have been proposed as targets for therapy (273). First, several miRNAs posttranscriptionally regulate genes associated with ROS production and inflammation. Thus, by regulating certain miRNAs we could reduce ROS and inflammation (274). Second, increased ROS levels could beneficial effects by activating apoptosis in response to chemical drugs or ionized radiation (275). In this line, ROS activates p53 and p38 MAPK making cancer cells more susceptible to treatment (275). Therefore, the use of drugs that promote the controlled formation of ROS could increase the efficacy of conventional therapies such as chemotherapy and radiotherapy (276).

A study of Temiz et al. reported that it is possible to decrease the expression of Chaperonin Containing TCP1 Subunit 3 (CCT3, protein essential in the folding of proteins involved in cell division, proliferation, and apoptosis) by transfecting mimics of miR-24-3p, miR-128-3p, and miR-149-5p in the BC cell line CRL-2329. The mimics promoted apoptosis by causing an imbalance in the intracellular ROS levels (277). Recently, Shang et al. transfected MDA-MB-231 cells with nanoparticles composed of chlorin e6 (Ce6)-anti-miR-21, Ce6-anti-miR-155 and zeolitic imidazolate framework-90 (ZIF-90) and observed a reduction of both miR-21 and miR-155, whereas the photosensitizer Ce6 induced the formation of ROS (278).

The development of resistance to chemotherapy is quite common in BC, and miRNAs are involved in this process. For example, the overexpression of miR-302b reduces cell viability and cell proliferation, making BC cells more sensitive to cisplatin. This miRNA regulates E2F transcription factor 1 (E2F1), a controller of the G1/S transition (279). Also, miR-125b regulates the resistance to doxorubicin by decreasing the mitochondrial membrane potential (MMP), downregulating the HS-1-associated protein X-1 (HAX-1), and releasing ROS from the mitochondria into the cytoplasm (280). On the other hand, overexpression of miR-24 induces resistance to cisplatin by regulating BCL2 like 11 (BimL), a pro-apoptotic factor in BC cells (281). In addition, miR-24 binds to the mRNA of *FIH1* and reduce its expression. Reduction of FIH1 increased the expression of HIF-1α, growth of breast cancer stem cells, and promoted the chemotherapy resistance (281). MiR-668 is overexpressed in BC cells that are resistant to radiotherapy. This miRNA acts on IκBα, promoting the activation of NF-kB (282). The expression of miR-223 in conjunction with TNF-related apoptosis-inducing ligand (TRAIL) in the MDA-MB-231 cell line caused an increase in ROS levels due to its action on the proto-oncogene *HAX-1*, making the cells more sensitive to doxorubicin and cisplatin (283). Yadav et al. found that the overexpression of miR-5096 downregulates the Solute Carrier Family Seven Number 11 (SLC7A11), a protein related to reducing ROS levels, in the MDA-MB-231 cell line, suggesting that miR-5096 has tumor suppressive roles (284). Wu et al. performed RNA and miRNAs sequencing studies with RNA extracted of healthy and tumor tissues of the breast, and by using bioinformatics found 219 miRNAs related to the ferroptosis, an iron-dependent cell death mechanism, characterized by the intracellular accumulation of ROS (285). All of these miRNAs could be used as targets for BC therapy. Reduced levels of these miRNAs will increase their targets genes, will reduce the ROS production, and will reduce the inflammation associated with BC progression.

## Conclusion

The production and regulation of ROS and miRNAs are interconnected each other, both being key regulators of inflammatory processes. Deregulation of these pathways promotes carcinogenesis in most cancer types, including BC. When conditions for oxidative stress exist, ROS overproduction can disturb the levels of various miRNAs. At the same time, deregulated miRNAs will alter ROS production. Alterations in cell homeostasis triggered by inflammatory processes facilitate the progression of BC by inducing processes such as migration, invasion, angiogenesis, drug resistance and/or inhibition of apoptosis. Better knowledge of the mechanisms involved in the regulation of these molecules it will allow to explore new approaches in the treatment of BC.

## Author contributions

Conceptualization PV-M and VG-V. Equal contribution and first authorship: VV-G and JE-J wrote the manuscript draft and elaborated the figures and tables. Review and editing: JV-G, DR-P, PV-M, and VG-V. All authors have read and agreed to the final version of the manuscript.

## Funding

This work was partially supported by the “Fondo Sectorial de Investigación para la Educación” 659 CB2017-2018. Programa Presupuestario F003 of the Consejo Nacional de Ciencia y Tecnología (CONACYT) (grant number A1-S-660 45974) (VG-V), the National Institute on Minority Health and Health Disparities (NIMHD) CCRHD 661 (U54MD007600) (PV-M), and by institutional seed funds from the UPR Comprehensive Cancer 662 Center (PV-M).

## Acknowledgments

We thank Alejandra Arreola-Triana for her support on editing this manuscript.

## Conflict of interest

The authors declare that the research was conducted in the absence of any commercial or financial relationships that could be construed as a potential conflict of interest.

## Publisher’s note

All claims expressed in this article are solely those of the authors and do not necessarily represent those of their affiliated organizations, or those of the publisher, the editors and the reviewers. Any product that may be evaluated in this article, or claim that may be made by its manufacturer, is not guaranteed or endorsed by the publisher.

## References

[1] Sung H Ferlay J Siegel RL Laversanne M Soerjomataram I Jemal A. Global cancer statistics 2020: GLOBOCAN estimates of incidence and mortality worldwide for 36 cancers in 185 countries. CA: A Cancer J Clin (2021) 71(3):209–49. doi: 10.3322/caac.21660 33538338

[2] Danaei G Hoorn SV Lopez AD Murray CJ Ezzati M. Causes of cancer in the world: comparative risk assessment of nine behavioural and environmental risk factors. Lancet (2005) 366:1784–93. doi: 10.1016/S0140-6736(05)67725-2 16298215

[3] Beral V Million Women Study Collaborators. Breast cancer and hormone-replacement therapy in the million women study. Lancet (2003) 362:419–27. doi: 10.1016/s0140-6736(03)14065-2 12927427

[4] American Cancer Society. Cancer facts & (022). Available at: https://www.cancer.org/research/cancer-facts-statistics/all-cancer-facts-figures/cancer-facts-figures-2022.html (Accessed June 15, 2022).

[5] Ahmad A ed. Breast cancer metastasis and drug resistance: Challenges and progress. Cham: Springer International Publishing (2019). doi: 10.1007/978-3-030-20301-6

[6] Weber GF. Molecular mechanisms of metastasis. Cancer Lett (2008) 270:181–90. doi: 10.1016/j.canlet.2008.04.030 18522865

[7] Ben-Baruch A. Host microenvironment in breast cancer development: Inflammatory cells, cytokines and chemokines in breast cancer progression: reciprocal tumor–microenvironment interactions. Breast Cancer Res (2003) 5:31–6. doi: 10.1186/bcr554 PMC154133 12559043

[8] Philip M Rowley DA Schreiber H. Inflammation as a tumor promoter in cancer induction. Semin Cancer Biol (2004) 14:433–9. doi: 10.1016/j.semcancer.2004.06.006 15489136

[9] DeNardo DG Coussens LM. Inflammation and breast cancer. balancing immune response: crosstalk between adaptive and innate immune cells during breast cancer progression. Breast Cancer Res (2007) 9:212. doi: 10.1186/bcr1746 17705880 PMC2206719

[10] Bahiraee A Ebrahimi R Halabian R Aghabozorgi AS Amani J. The role of inflammation and its related microRNAs in breast cancer: A narrative review. J Cell Physiol (2019) 234:19480–93. doi: 10.1002/jcp.28742 31025369

[11] Allen MD Jones LJ. The role of inflammation in progression of breast cancer: Friend or foe? Int J Oncol (2015) 47:797–805. doi: 10.3892/ijo.2015.3075 26165857

[12] Snezhkina AV Kudryavtseva AV Kardymon OL Savvateeva MV Melnikova NV Krasnov GS. ROS generation and antioxidant defense systems in normal and malignant cells. Oxid Med Cell Longevity (2019) 2019:e6175804. doi: 10.1155/2019/6175804 PMC6701375 31467634

[13] He J Jiang B-H. Interplay between reactive oxygen species and MicroRNAs in cancer. Curr Pharmacol Rep (2016) 2:82–90. doi: 10.1007/s40495-016-0051-4 27284501 PMC4894750

[14] Brieger K Schiavone S Miller FJ Krause K-H. Reactive oxygen species: From health to disease. Swiss Med Weekly (2012) 142:1–14. doi: 10.4414/smw.2012.13659 22903797

[15] Liou G-Y Storz P. Reactive oxygen species in cancer. Free Radical Res (2010) 44:479–96. doi: 10.3109/10715761003667554 PMC3880197 20370557

[16] Seyfried TN Huysentruyt LC. On the origin of cancer metastasis. CRO (2013) 18:43–73. doi: 10.1615/CritRevOncog.v18.i1-2.40 PMC3597235 23237552

[17] Cosentino G Plantamura I Cataldo A Iorio MV. MicroRNA and oxidative stress interplay in the context of breast cancer pathogenesis. Int J Mol Sci (2019) 20:e5143. doi: 10.3390/ijms20205143 31627322 PMC6829356

[18] Hong S Noh H Teng Y Shao J Rehmani H Ding H-F. SHOX2 is a direct miR-375 target and a novel epithelial-to-mesenchymal transition inducer in breast cancer cells. Neoplasia (2014) 16:279–90. doi: 10.1016/j.neo.2014.03.010 PMC4094831 24746361

[19] Loh H-Y Norman BP Lai K-S NMANAbd R Alitheen NBM Osman MA. The regulatory role of MicroRNAs in breast cancer. Int J Mol Sci (2019) 20:1–27. doi: 10.3390/ijms20194940 PMC6801796 31590453

[20] Volovat SR Volovat C Hordila I Hordila D-A Mirestean CC Miron OT. MiRNA and LncRNA as potential biomarkers in triple-negative breast cancer: A review. Front Oncol (2020) 10:526850. doi: 10.3389/fonc.2020.526850 33330019 PMC7716774

[21] Babu K R Tay Y. The yin-yang regulation of reactive oxygen species and MicroRNAs in cancer. Int J Mol Sci (2019) 20:1–21. doi: 10.3390/ijms20215335 PMC6862169 31717786

[22] Lan J Huang Z Han J Shao J Huang C. Redox regulation of microRNAs in cancer. Cancer Lett (2018) 418:250–9. doi: 10.1016/j.canlet.2018.01.010 29330105

[23] Provenzano E Ulaner GA Chin S-F. Molecular classification of breast cancer. PET Clinics (2018) 13:325–38. doi: 10.1016/j.cpet.2018.02.004 30100073

[24] Genome-driven integrated classification of breast cancer validated in over 7,500 samples, in: Genome biology Available at: https://genomebiology.biomedcentral.com/articles (Accessed April 25, 2021). 10.1186/s13059-014-0431-1 PMC4166472 25164602

[25] IARC publications website - WHO classification of tumours. Available at: https://publications.iarc.fr/Book-And-Report-Series/Who-Classification-Of-Tumours (Accessed April 25, 2021).

[26] Tsang JYS Tse GM. Molecular classification of breast cancer. Adv Anatomic Pathol (2020) 27:27–35. doi: 10.1097/PAP.0000000000000232 31045583

[27] Mook S Schmidt MK Rutgers EJ van de Velde AO Visser O Rutgers SM. Calibration and discriminatory accuracy of prognosis calculation for breast cancer with the online adjuvant! program: a hospital-based retrospective cohort study. Lancet Oncol (2009) 10:1070–6. doi: 10.1016/S1470-2045(09)70254-2 19801202

[28] Weiss A Chavez-MacGregor M Lichtensztajn DY Yi M Tadros A Hortobagyi GN. Validation study of the American joint committee on cancer eighth edition prognostic stage compared with the anatomic stage in breast cancer. JAMA Oncol (2018) 4:203–9. doi: 10.1001/jamaoncol.2017.4298 PMC5838705 29222540

[29] van ‘t Veer LJ Dai H van de Vijver MJ He YD Hart AAM Mao M. Gene expression profiling predicts clinical outcome of breast cancer. Nature (2002) 415:530–6. doi: 10.1038/415530a 11823860

[30] Sørlie T Perou CM Tibshirani R Aas T Geisler S Johnsen H. Gene expression patterns of breast carcinomas distinguish tumor subclasses with clinical implications. Proc Natl Acad Sci USA (2001) 98:10869–74. doi: 10.1073/pnas.191367098 PMC58566 11553815

[31] Tsang JYS Tse GM. Molecular classification of breast cancer. Adv Anatomic Pathol (2020) 27:27–35. doi: 10.1097/PAP.0000000000000232 31045583

[32] Watkins EJ. Overview of breast cancer. J Am Acad PAs (2019) 32:13–7. doi: 10.1097/01.JAA.0000580524.95733.3d 31513033

[33] Merino Bonilla JA Torres Tabanera M Ros Mendoza LH. Breast cancer in the 21st century: from early detection to new therapies. Radiologia (2017) 59:368–79. doi: 10.1016/j.rx.2017.06.003 28712528

[34] FI G EM S ID G MTP S CF Z. Clasificación molecular del cáncer de mama. Cuadernos Cirugía (2018) 25:67–74. doi: 10.4206/cuad.cir.2011.v25n1-10

[35] Yin L Duan J-J Bian X-W Yu S. Triple-negative breast cancer molecular subtyping and treatment progress. Breast Cancer Res (2020) 22:61. doi: 10.1186/s13058-020-01296-5 32517735 PMC7285581

[36] Dai X Li T Bai Z Yang Y Liu X Zhan J. Breast cancer intrinsic subtype classification, clinical use and future trends. Am J Cancer Res (2015) 5:2929–43. PMC4656721 26693050

[37] Perou CM Sørlie T Eisen MB van de Rijn M Jeffrey SS Rees CA. Molecular portraits of human breast tumours. Nature (2000) 406:747–52. doi: 10.1038/35021093 10963602

[38] Voduc KD Cheang MCU Tyldesley S Gelmon K Nielsen TO Kennecke H. Breast cancer subtypes and the risk of local and regional relapse. J Clin Oncol (2010) 28:1684–91. doi: 10.1200/JCO.2009.24.9284 20194857

[39] Weigelt B Baehner FL Reis-Filho JS. The contribution of gene expression profiling to breast cancer classification, prognostication and prediction: a retrospective of the last decade. J Pathol (2010) 220:263–80. doi: 10.1002/path.2648 19927298

[40] Ades F Zardavas D Bozovic-Spasojevic I Pugliano L Fumagalli D de Azambuja E. Luminal b breast cancer: molecular characterization, clinical management, and future perspectives. J Clin Oncol (2014) 32:2794–803. doi: 10.1200/JCO.2013.54.1870 25049332

[41] de Azambuja E Cardoso F de Castro G Colozza M Mano MS Durbecq V. Ki-67 as prognostic marker in early breast cancer: a meta-analysis of published studies involving 12,155 patients. Br J Cancer (2007) 96:1504–13. doi: 10.1038/sj.bjc.6603756 PMC2359936 17453008

[42] Trihia H Murray S Price K Gelber RD Golouh R Goldhirsch A. Ki-67 expression in breast carcinoma: its association with grading systems, clinical parameters, and other prognostic factors–a surrogate marker? Cancer (2003) 97:1321–31. doi: 10.1002/cncr.11188 12599241

[43] Hashmi AA Hashmi KA Irfan M Khan SM Edhi MM Ali JP. Ki67 index in intrinsic breast cancer subtypes and its association with prognostic parameters. BMC Res Notes (2019) 12:605. doi: 10.1186/s13104-019-4653-x 31547858 PMC6755684

[44] Cheang MCU Chia SK Voduc D Gao D Leung S Snider J. Ki67 index, HER2 status, and prognosis of patients with luminal b breast cancer. J Natl Cancer Inst (2009) 101:736–50. doi: 10.1093/jnci/djp082 PMC2684553 19436038

[45] Parker JS Mullins M Cheang MCU Leung S Voduc D Vickery T. Supervised risk predictor of breast cancer based on intrinsic subtypes. J Clin Oncol (2009) 27:1160–7. doi: 10.1200/JCO.2008.18.1370 PMC2667820 19204204

[46] Lehmann BD Bauer JA Chen X Sanders ME Chakravarthy AB Shyr Y. Identification of human triple-negative breast cancer subtypes and preclinical models for selection of targeted therapies. J Clin Invest (2011) 121:2750–67. doi: 10.1172/JCI45014 PMC3127435 21633166

[47] Lehmann BD Jovanović B Chen X Estrada MV Johnson KN Shyr Y. Refinement of triple-negative breast cancer molecular subtypes: Implications for neoadjuvant chemotherapy selection. PLoS One (2016) 11:e0157368. doi: 10.1371/journal.pone.0157368 27310713 PMC4911051

[48] Masuda H Baggerly KA Wang Y Zhang Y Gonzalez-Angulo AM Meric-Bernstam F. Differential response to neoadjuvant chemotherapy among 7 triple-negative breast cancer molecular subtypes. Clin Cancer Res (2013) 19:5533–40. doi: 10.1158/1078-0432.CCR-13-0799 PMC3813597 23948975

[49] Badve S Dabbs DJ Schnitt SJ Baehner FL Decker T Eusebi V. Basal-like and triple-negative breast cancers: a critical review with an emphasis on the implications for pathologists and oncologists. Mod Pathol (2011) 24:157–67. doi: 10.1038/modpathol.2010.200 21076464

[50] Peppercorn J Perou CM Carey LA. Molecular subtypes in breast cancer evaluation and management: divide and conquer. Cancer Invest (2008) 26:1–10. doi: 10.1080/07357900701784238 18181038

[51] van Uden DJP van Laarhoven HWM Westenberg AH de Wilt JHW Blanken-Peeters CFJM. Inflammatory breast cancer: An overview. Crit Rev Oncology/Hematology (2015) 93:116–26. doi: 10.1016/j.critrevonc.2014.09.003 25459672

[52] Lim B Woodward WA Wang X Reuben JM Ueno NT. Inflammatory breast cancer biology: the tumour microenvironment is key. Nat Rev Cancer (2018) 18:485–99. doi: 10.1038/s41568-018-0010-y 29703913

[53] Zell JA Tsang WY Taylor TH Mehta RS Anton-Culver H. Prognostic impact of human epidermal growth factor-like receptor 2 and hormone receptor status in inflammatory breast cancer (IBC): analysis of 2,014 IBC patient cases from the California cancer registry. Breast Cancer Res (2009) 11:R9. doi: 10.1186/bcr2225 19228416 PMC2687713

[54] Rosenbluth JM Overmoyer BA. Inflammatory breast cancer: a separate entity. Curr Oncol Rep (2019) 21:86. doi: 10.1007/s11912-019-0842-y 31414257

[55] Sobin LH Compton CC. TNM seventh edition: what’s new, what’s changed: communication from the international union against cancer and the American joint committee on cancer. Cancer (2010) 116:5336–9. doi: 10.1002/cncr.25537 20665503

[56] Jolly MK Boareto M Debeb BG Aceto N Farach-Carson MC Woodward WA. Inflammatory breast cancer: a model for investigating cluster-based dissemination. NPJ Breast Cancer (2017) 3:1–8. doi: 10.1038/s41523-017-0023-9 28649661 PMC5460282

[57] Marotta LLC Almendro V Marusyk A Shipitsin M Schemme J Walker SR. The JAK2/STAT3 signaling pathway is required for growth of CD44^+^CD24^–^ stem cell–like breast cancer cells in human tumors. J Clin Invest (2011) 121:2723–35. doi: 10.1172/JCI44745 PMC3223826 21633165

[58] Medzhitov R. Origin and physiological roles of inflammation. Nature (2008) 454:428–35. doi: 10.1038/nature07201 18650913

[59] Serhan CN Savill J. Resolution of inflammation: the beginning programs the end. Nat Immunol (2005) 6:1191–7. doi: 10.1038/ni1276 16369558

[60] Furman D Campisi J Verdin E Carrera-Bastos P Targ S Franceschi C. Chronic inflammation in the etiology of disease across the life span. Nat Med (2019) 25:1822–32. doi: 10.1038/s41591-019-0675-0 PMC7147972 31806905

[61] Grivennikov SI Greten FR Karin M. Immunity, inflammation, and cancer. Cell (2010) 140:883–99. doi: 10.1016/j.cell.2010.01.025 PMC2866629 20303878

[62] Fridman WH Zitvogel L Sautès–Fridman C Kroemer G. The immune contexture in cancer prognosis and treatment. Nat Rev Clin Oncol (2017) 14:717–34. doi: 10.1038/nrclinonc.2017.101 28741618

[63] Condeelis J Pollard JW. Macrophages: Obligate partners for tumor cell migration, invasion, and metastasis. Cell (2006) 124:263–6. doi: 10.1016/j.cell.2006.01.007 16439202

[64] Murdoch C Muthana M Coffelt SB Lewis CE. The role of myeloid cells in the promotion of tumour angiogenesis. Nat Rev Cancer (2008) 8:618–31. doi: 10.1038/nrc2444 18633355

[65] Sica A Mantovani A. Macrophage plasticity and polarization: *in vivo* veritas. J Clin Invest (2012) 122:787–95. doi: 10.1172/JCI59643 PMC3287223 22378047

[66] Sica A Larghi P Mancino A Rubino L Porta C Totaro MG. Macrophage polarization in tumour progression. Semin Cancer Biol (2008) 18:349–55. doi: 10.1016/j.semcancer.2008.03.004 18467122

[67] Murray PJ. Macrophage polarization. Annu Rev Physiol (2017) 79:541–66. doi: 10.1146/annurev-physiol-022516-034339 27813830

[68] Bashir S Sharma Y Elahi A Khan F. Macrophage polarization: the link between inflammation and related diseases. Inflammation Res (2016) 65:1–11. doi: 10.1007/s00011-015-0874-1 26467935

[69] Wang N Liang H Zen K. Molecular mechanisms that influence the macrophage M1–M2 polarization balance. Front Immunol (2014) 5:614. doi: 10.3389/fimmu.2014.00614 25506346 PMC4246889

[70] Jetten N Verbruggen S Gijbels MJ Post MJ De Winther MPJ Donners MMPC. Anti-inflammatory M2, but not pro-inflammatory M1 macrophages promote angiogenesis. vivo. Angiogenesis (2014) 17:109–18. doi: 10.1007/s10456-013-9381-6 24013945

[71] Ruffell B Affara NI Coussens LM. Differential macrophage programming in the tumor microenvironment. Trends Immunol (2012) 33:119–26. doi: 10.1016/j.it.2011.12.001 PMC3294003 22277903

[72] Qian B-Z Pollard JW. Macrophage diversity enhances tumor progression and metastasis. Cell (2010) 141:39–51. doi: 10.1016/j.cell.2010.03.014 20371344 PMC4994190

[73] Pollard JW. Tumour-educated macrophages promote tumour progression and metastasis. Nat Rev Cancer (2004) 4:71–8. doi: 10.1038/nrc1256 14708027

[74] Malyshev I Malyshev Y. Current concept and update of the macrophage plasticity concept: Intracellular mechanisms of reprogramming and M3 macrophage “Switch” phenotype. BioMed Res Int (2015) 2015:341308. doi: 10.1155/2015/341308 26366410 PMC4561113

[75] Malyshev I Lyamina S. Imbalance of M1/M2 alveolar macrophages phenotype in bronchial asthma (LB506). FASEB J (2014) 28:LB506. doi: 10.1096/fasebj.28.1_supplement.lb506

[76] Lyamina S Malyshev I. Imbalance of immune response functional phenotype and alveolar macrophages phenotype in COPD(2014). Available at: https://erj.ersjournals.com/content/44/Suppl_58/P1483 (Accessed July 26, 2022).

[77] Schleimer RP. Effects of glucocorticosteroids on inflammatory cells relevant to their therapeutic applications in asthma. Am Rev Respir Dis (1990) 141:S59–69. 2178515

[78] Jackaman C Yeoh TL Acuil ML Gardner JK Nelson DJ. Murine mesothelioma induces locally-proliferating IL-10+TNF-α+CD206–CX3CR1+ M3 macrophages that can be selectively depleted by chemotherapy or immunotherapy. Oncoimmunology (2016) 5:e1173299. doi: 10.1080/2162402X.2016.1173299 27471652 PMC4938311

[79] Kalish S Lyamina S Manukhina E Malyshev Y Raetskaya A Malyshev I. M3 macrophages stop division of tumor cells *In vitro* and extend survival of mice with Ehrlich ascites carcinoma. Med Sci Monit Basic Res (2017) 23:8–19. doi: 10.12659/MSMBR.902285 28123171 PMC5291087

[80] Kolaczkowska E Kubes P. Neutrophil recruitment and function in health and inflammation. Nat Rev Immunol (2013) 13:159–75. doi: 10.1038/nri3399 23435331

[81] Hinshaw DC Shevde LA. The tumor microenvironment innately modulates cancer progression. Cancer Res (2019) 79:4557–66. doi: 10.1158/0008-5472.CAN-18-3962 PMC6744958 31350295

[82] Fridlender ZG Sun J Kim S Kapoor V Cheng G Ling L. Polarization of tumor-associated neutrophil (TAN) phenotype by TGF-β: “N1” versus “N2” TAN. Cancer Cell (2009) 16:183–94. doi: 10.1016/j.ccr.2009.06.017 PMC2754404 19732719

[83] Hajizadeh F Aghebati Maleki L Alexander M Mikhailova MV Masjedi A Ahmadpour M. Tumor-associated neutrophils as new players in immunosuppressive process of the tumor microenvironment in breast cancer. Life Sci (2021) 264:118699. doi: 10.1016/j.lfs.2020.118699 33137368

[84] Antonio N Bønnelykke-Behrndtz ML Ward LC Collin J Christensen IJ Steiniche T. The wound inflammatory response exacerbates growth of pre-neoplastic cells and progression to cancer. EMBO J (2015) 34:2219–36. doi: 10.15252/embj.201490147 PMC4585460 26136213

[85] Anderson NM Simon MC. Tumor microenvironment. Curr Biol (2020) 30:R921–5. doi: 10.1016/j.cub.2020.06.081 PMC8194051 32810447

[86] Demers M Wong SL Martinod K Gallant M Cabral JE Wang Y. Priming of neutrophils toward NETosis promotes tumor growth. Oncoimmunology (2016) 5:e1134073. doi: 10.1080/2162402X.2015.1134073 27467952 PMC4910712

[87] Jamieson T Clarke M Steele CW Samuel MS Neumann J Jung A. Inhibition of CXCR2 profoundly suppresses inflammation-driven and spontaneous tumorigenesis. J Clin Invest (2012) 122:3127–44. doi: 10.1172/JCI61067 PMC3428079 22922255

[88] Sharma B Nawandar DM Nannuru KC Varney ML Singh RK. Targeting CXCR2 enhances chemotherapeutic response, inhibits mammary tumor growth, angiogenesis and lung metastasis. Mol Cancer Ther (2013) 12:10. doi: 10.1158/1535-7163.MCT-12-0529 PMC3653628 23468530

[89] Queen MM Ryan RE Holzer RG Keller-Peck CR Jorcyk CL. Breast cancer cells stimulate neutrophils to produce oncostatin m: potential implications for tumor progression. Cancer Res (2005) 65:8896–904. doi: 10.1158/0008-5472.CAN-05-1734 16204061

[90] Wu L Awaji M Saxena S Varney ML Sharma B Singh RK. IL-17–CXC chemokine receptor 2 axis facilitates breast cancer progression by up-regulating neutrophil recruitment. Am J Pathol (2020) 190:222–33. doi: 10.1016/j.ajpath.2019.09.016 PMC6943375 31654638

[91] Innate immune cells in the tumor microenvironment, in: Cancer cell . Available at: https://www.cell.com/cancer-cell/fulltext/S1535-6108(21)00282-8 (Accessed July 24, 2022). 10.1016/j.ccell.2021.05.016 34129817

[92] Shi H Zhang J Han X Li H Xie M Sun Y. Recruited monocytic myeloid-derived suppressor cells promote the arrest of tumor cells in the premetastatic niche through an IL-1β-mediated increase in e-selectin expression. Int J Cancer (2017) 140:1370–83. doi: 10.1002/ijc.30538 27885671

[93] Bates JP Derakhshandeh R Jones L Webb TJ. Mechanisms of immune evasion in breast cancer. BMC Cancer (2018) 18:556. doi: 10.1186/s12885-018-4441-3 29751789 PMC5948714

[94] Condamine T Ramachandran I Youn J-I Gabrilovich DI. Regulation of tumor metastasis by myeloid-derived suppressor cells. Annu Rev Med (2015) 66:97–110. doi: 10.1146/annurev-med-051013-052304 25341012 PMC4324727

[95] Diaz-Montero CM Salem ML Nishimura MI Garrett-Mayer E Cole DJ Montero AJ. Increased circulating myeloid-derived suppressor cells correlate with clinical cancer stage, metastatic tumor burden, and doxorubicin–cyclophosphamide chemotherapy. Cancer Immunol Immunother (2009) 58:49–59. doi: 10.1007/s00262-008-0523-4 18446337 PMC3401888

[96] Qian B-Z Li J Zhang H Kitamura T Zhang J Campion LR. CCL2 recruits inflammatory monocytes to facilitate breast-tumour metastasis. Nature (2011) 475:222–5. doi: 10.1038/nature10138 PMC3208506 21654748

[97] DeNardo DG Barreto JB Andreu P Vasquez L Tawfik D Kolhatkar N. CD4(+) T cells regulate pulmonary metastasis of mammary carcinomas by enhancing protumor properties of macrophages. Cancer Cell (2009) 16:91–102. doi: 10.1016/j.ccr.2009.06.018 19647220 PMC2778576

[98] Mj S Gp D Rd S. Cancer immunosurveillance and immunoediting: the roles of immunity in suppressing tumor development and shaping tumor immunogenicity. Adv Immunol (2006) 90:1–50. doi: 10.1016/S0065-2776(06)90001-7 16730260

[99] Adams EJ Gu S Luoma AM. Human gamma delta T cells: evolution and ligand recognition. Cell Immunol (2015) 296:31–40. doi: 10.1016/j.cellimm.2015.04.008 25991474 PMC4466157

[100] Chabab G Barjon C Abdellaoui N Salvador-Prince L Dejou C Michaud H-A. Identification of a regulatory Vδ1 gamma delta T cell subpopulation expressing CD73 in human breast cancer. J Leukocyte Biol (2020) 107:1057–67. doi: 10.1002/JLB.3MA0420-278RR 32362028

[101] Kuzet S-E Gaggioli C. Fibroblast activation in cancer: when seed fertilizes soil. Cell Tissue Res (2016) 365:607–19. doi: 10.1007/s00441-016-2467-x 27474009

[102] Annaratone L Cascardi E Vissio E Sarotto I Chmielik E Sapino A. The multifaceted nature of tumor microenvironment in breast carcinomas. PAT (2020) 87:125–42. doi: 10.1159/000507055 PMC7265767 32325459

[103] Gascard P Tlsty TD. Carcinoma-associated fibroblasts: orchestrating the composition of malignancy. Genes Dev (2016) 30:1002–19. doi: 10.1101/gad.279737.116 PMC4863733 27151975

[104] Kessenbrock K Plaks V Werb Z. Matrix metalloproteinases: Regulators of the tumor microenvironment. Cell (2010) 141:52–67. doi: 10.1016/j.cell.2010.03.015 20371345 PMC2862057

[105] Li H Qiu Z Li F Wang C. The relationship between MMP-2 and MMP-9 expression levels with breast cancer incidence and prognosis. Oncol Lett (2017) 14:5865–70. doi: 10.3892/ol.2017.6924 PMC5661385 29113219

[106] Mehner C Hockla A Miller E Ran S Radisky DC Radisky ES. Tumor cell-produced matrix metalloproteinase 9 (MMP-9) drives malignant progression and metastasis of basal-like triple negative breast cancer. Oncotarget (2014) 5:2736–49. doi: 10.18632/oncotarget.1932 PMC4058041 24811362

[107] Wu Q Li B Li Z Li J Sun S Sun S. Cancer-associated adipocytes: key players in breast cancer progression. J Hematol Oncol (2019) 12:95. doi: 10.1186/s13045-019-0778-6 31500658 PMC6734503

[108] Choi J Cha YJ Koo JS. Adipocyte biology in breast cancer: From silent bystander to active facilitator. Prog Lipid Res (2018) 69:11–20. doi: 10.1016/j.plipres.2017.11.002 29175445

[109] He J-Y Wei X-H Li S-J Liu Y Hu H-L Li Z-Z. Adipocyte-derived IL-6 and leptin promote breast cancer metastasis *via* upregulation of lysyl hydroxylase-2 expression. Cell Communication Signaling (2018) 16:100. doi: 10.1186/s12964-018-0309-z 30563531 PMC6299564

[110] Gyamfi J Eom M Koo J-S Choi J. Multifaceted roles of interleukin-6 in adipocyte–breast cancer cell interaction. Trans Oncol (2018) 11:275–85. doi: 10.1016/j.tranon.2017.12.009 PMC5884177 29413760

[111] Ben-Baruch A. Host microenvironment in breast cancer development: Inflammatory cells, cytokines and chemokines in breast cancer progression: reciprocal tumor–microenvironment interactions. Breast Cancer Res (2003) 5:31–6. doi: 10.1186/bcr554 PMC154133 12559043

[112] Colotta F Allavena P Sica A Garlanda C Mantovani A. Cancer-related inflammation, the seventh hallmark of cancer: links to genetic instability. Carcinogenesis (2009) 30:1073–81. doi: 10.1093/carcin/bgp127 19468060

[113] Grivennikov SI Greten FR Karin M. Immunity, inflammation, and cancer. Cell (2010) 140:883–99. doi: 10.1016/j.cell.2010.01.025 PMC2866629 20303878

[114] Barcellos-Hoff MH Akhurst RJ. Transforming growth factor-β in breast cancer: too much, too late. Breast Cancer Res (2009) 11:202. doi: 10.1186/bcr2224 19291273 PMC2687712

[115] Dongwu L Xiaoqian W Zhiwei C. Tumor necrosis factor-α, a regulator and therapeutic agent on breast cancer. Curr Pharm Biotechnol (2016) 17:486–94. doi: 10.2174/1389201017666160301102713 26927216

[116] Martínez-Reza I Díaz L García-Becerra R. Preclinical and clinical aspects of TNF-α and its receptors TNFR1 and TNFR2 in breast cancer. J Biomed Sci (2017) 24:90. doi: 10.1186/s12929-017-0398-9 29202842 PMC5713022

[117] Cruceriu D Baldasici O Balacescu O Berindan-Neagoe I. The dual role of tumor necrosis factor-alpha (TNF-α) in breast cancer: molecular insights and therapeutic approaches. Cell Oncol (2020) 43:1–18. doi: 10.1007/s13402-019-00489-1 PMC12990688 31900901

[118] Deshmukh SK Srivastava SK Bhardwaj A Singh AP Tyagi N Marimuthu S. Resistin and interleukin-6 exhibit racially-disparate expression in breast cancer patients, display molecular association and promote growth and aggressiveness of tumor cells through STAT3 activation. Oncotarget (2015) 6:11231–41. doi: 10.18632/oncotarget.3591 PMC4484452 25868978

[119] Deshmukh SK Srivastava SK Zubair H Bhardwaj A Tyagi N Al-Ghadhban A. Resistin potentiates chemoresistance and stemness of breast cancer cells: Implications for racially disparate therapeutic outcomes. Cancer Lett (2017) 396:21–9. doi: 10.1016/j.canlet.2017.03.010 PMC5437742 28302531

[120] Lee Y-C Chen Y-J Wu C-C Lo S Hou M-F Yuan S-SF. Resistin expression in breast cancer tissue as a marker of prognosis and hormone therapy stratification. Gynecol Oncol (2012) 125:742–50. doi: 10.1016/j.ygyno.2012.02.032 22370603

[121] Esquivel-Velázquez M Ostoa-Saloma P Palacios-Arreola MI Nava-Castro KE Castro JI Morales-Montor J. The role of cytokines in breast cancer development and progression. J Interferon Cytokine Res (2014) 35:1–16. doi: 10.1089/jir.2014.0026 25068787 PMC4291218

[122] Knüpfer H Preiß R. Significance of interleukin-6 (IL-6) in breast cancer (review). Breast Cancer Res Treat (2007) 102:129–35. doi: 10.1007/s10549-006-9328-3 16927176

[123] Leu C-M Wong F-H Chang C Huang S-F Hu C. Interleukin-6 acts as an antiapoptotic factor in human esophageal carcinoma cells through the activation of both STAT3 and mitogen-activated protein kinase pathways. Oncogene (2003) 22:7809–18. doi: 10.1038/sj.onc.1207084 14586407

[124] Gyamfi J Eom M Koo J-S Choi J. Multifaceted roles of interleukin-6 in adipocyte–breast cancer cell interaction. Trans Oncol (2018) 11:275–85. doi: 10.1016/j.tranon.2017.12.009 PMC5884177 29413760

[125] Johnson DE O’Keefe RA Grandis JR. Targeting the IL-6/JAK/STAT3 signalling axis in cancer. Nat Rev Clin Oncol (2018) 15:234–48. doi: 10.1038/nrclinonc.2018.8 PMC5858971 29405201

[126] Li H Yang B Huang J Lin Y Xiang T Wan J. Cyclooxygenase-2 in tumor-associated macrophages promotes breast cancer cell survival by triggering a positive-feedback loop between macrophages and cancer cells. Oncotarget (2015) 6:29637–50. doi: 10.18632/oncotarget.4936 PMC4745752 26359357

[127] Eagar TN Miller SD Rich RR Fleisher TA Shearer WT Schroeder HW. “16 - helper T-cell subsets and control of the inflammatory response.,”. In: Clinical immunology, Fifth Edition, vol. London: Elsevier (2019). p. 235–245.e1. doi: 10.1016/B978-0-7020-6896-6.00016-8

[128] Zhao J Chen X Herjan T Li X. The role of interleukin-17 in tumor development and progression. J Exp Med (2019) 217:e20190297. doi: 10.1084/jem.20190297 PMC7037244 31727782

[129] Marcuzzi E Angioni R Molon B Calì B. Chemokines and chemokine receptors: Orchestrating tumor metastasization. Int J Mol Sci (2018) 20:96. doi: 10.3390/ijms20010096 PMC6337330 30591657

[130] Rizeq B Malki MI. The role of CCL21/CCR7 chemokine axis in breast cancer progression. Cancers (Basel) (2020) 12:1036. doi: 10.3390/cancers12041036 PMC7226115 32340161

[131] Kadomoto S Izumi K Mizokami A. The CCL20-CCR6 axis in cancer progression. Int J Mol Sci (2020) 21:5186. doi: 10.3390/ijms21155186 PMC7432448 32707869

[132] Lee SK Park K-K Kim H-J Park J Son SH Kim KR. Human antigen r-regulated CCL20 contributes to osteolytic breast cancer bone metastasis. Sci Rep (2017) 7:9610. doi: 10.1038/s41598-017-09040-4 28851919 PMC5575024

[133] Ha H Debnath B Neamati N. Role of the CXCL8-CXCR1/2 axis in cancer and inflammatory diseases. Theranostics (2017) 7:1543–88. doi: 10.7150/thno.15625 PMC5436513 28529637

[134] Fernando RI Castillo MD Litzinger M Hamilton DH Palena C. IL-8 signaling plays a critical role in the epithelial-mesenchymal transition of human carcinoma cells. Cancer Res (2011) 71:5296–306. doi: 10.1158/0008-5472.CAN-11-0156 PMC3148346 21653678

[135] Liubomirski Y Lerrer S Meshel T Rubinstein-Achiasaf L Morein D Wiemann S. Tumor-Stroma-Inflammation networks promote pro-metastatic chemokines and aggressiveness characteristics in triple-negative breast cancer. Front Immunol (2019) 10:757. doi: 10.3389/fimmu.2019.00757 31031757 PMC6473166

[136] Sharma B Nannuru KC Varney ML Singh RK. Host Cxcr2-dependent regulation of mammary tumor growth and metastasis. Clin Exp Metastasis (2015) 32:65–72. doi: 10.1007/s10585-014-9691-0 25511644 PMC4821540

[137] Zhou J Xiang Y Yoshimura T Chen K Gong W Huang J. The role of chemoattractant receptors in shaping the tumor microenvironment. BioMed Res Int (2014) 2014:e751392. doi: 10.1155/2014/751392 PMC4119707 25110692

[138] Bennett M Gilroy DW. Lipid mediators in inflammation. Microbiol Spectr (2016) 4:4.6.06. doi: 10.1128/microbiolspec.MCHD-0035-2016 27837747

[139] Smith WL DeWitt DL Garavito RM. Cyclooxygenases: structural, cellular, and molecular biology. Annu Rev Biochem (2000) 69:145–82. doi: 10.1146/annurev.biochem.69.1.145 10966456

[140] Finetti F Travelli C Ercoli J Colombo G Buoso E Trabalzini L. Prostaglandin E2 and cancer: Insight into tumor progression and immunity. Biol (Basel) (2020) 9:434. doi: 10.3390/biology9120434 PMC7760298 33271839

[141] Walker OL Dahn ML Power Coombs MR Marcato P. The prostaglandin E2 pathway and breast cancer stem cells: Evidence of increased signaling and potential targeting. Front Oncol (2022) 11:791696. doi: 10.3389/fonc.2021.791696 35127497 PMC8807694

[142] Ching MM Reader J Fulton AM. Eicosanoids in cancer: Prostaglandin E2 receptor 4 in cancer therapeutics and immunotherapy. Front Pharmacol (2020) 11:819. doi: 10.3389/fphar.2020.00819 32547404 PMC7273839

[143] Li S Xu X Jiang M Bi Y Xu J Han M. Lipopolysaccharide induces inflammation and facilitates lung metastasis in a breast cancer model *via* the prostaglandin E2-EP2 pathway. Mol Med Rep (2015) 11:4454–62. doi: 10.3892/mmr.2015.3258 25625500

[144] Robertson FM Simeone A-M Mazumdar A Shah AH McMurray JS Ghosh S. Molecular and pharmacological blockade of the EP4 receptor selectively inhibits both proliferation and invasion of human inflammatory breast cancer cells. J Exp Ther Oncol (2008) 7:299–312. 19227010

[145] Kochel TJ Goloubeva OG Fulton AM. Upregulation of cyclooxygenase-2/Prostaglandin E2 (COX-2/PGE2) pathway member multiple drug resistance-associated protein 4 (MRP4) and downregulation of prostaglandin transporter (PGT) and 15-prostaglandin dehydrogenase (15-PGDH) in triple-negative breast cancer. Breast Cancer (Auckl) (2016) 10:61–70. doi: 10.4137/BCBCR.S38529 27257388 PMC4881873

[146] Chen X Song M Zhang B Zhang Y. Reactive oxygen species regulate T cell immune response in the tumor microenvironment. Oxid Med Cell Longevity (2016) 2016:e1580967. doi: 10.1155/2016/1580967 PMC4980531 27547291

[147] Liou G-Y Storz P. Reactive oxygen species in cancer. Free Radical Res (2010) 44:479–96. doi: 10.3109/10715761003667554 PMC3880197 20370557

[148] Dröge W. Free radicals in the physiological control of cell function. Physiol Rev (2002) 82:47–95. doi: 10.1152/physrev.00018.2001 11773609

[149] Holmström KM Finkel T. Cellular mechanisms and physiological consequences of redox-dependent signalling. Nat Rev Mol Cell Biol (2014) 15:411–21. doi: 10.1038/nrm3801 24854789

[150] Medzhitov R. Origin and physiological roles of inflammation. Nature (2008) 454:428–35. doi: 10.1038/nature07201 18650913

[151] Mittal M Siddiqui MR Tran K Reddy SP Malik AB. Reactive oxygen species in inflammation and tissue injury. Antioxid Redox Signal (2014) 20:1126–67. doi: 10.1089/ars.2012.5149 PMC3929010 23991888

[152] Hurd TR DeGennaro M Lehmann R. Redox regulation of cell migration and adhesion. Trends Cell Biol (2012) 22:107–15. doi: 10.1016/j.tcb.2011.11.002 PMC4515034 22209517

[153] Bedard K Krause K-H. The NOX family of ROS-generating NADPH oxidases: Physiology and pathophysiology. Physiol Rev (2007) 87:245–313. doi: 10.1152/physrev.00044.2005 17237347

[154] Reuter S Gupta SC Chaturvedi MM Aggarwal BB. Oxidative stress, inflammation, and cancer: How are they linked? Free Radical Biol Med (2010) 49:1603–16. doi: 10.1016/j.freeradbiomed.2010.09.006 PMC2990475 20840865

[155] Wu Y Antony S Meitzler JL Doroshow JH. Molecular mechanisms underlying chronic inflammation-associated cancers. Cancer Lett (2014) 345:164–73. doi: 10.1016/j.canlet.2013.08.014 PMC3935998 23988267

[156] Kashyap D Tuli HS Sak K Garg VK Goel N Punia S. Role of reactive oxygen species in cancer progression. Curr Pharmacol Rep (2019) 5:79–86. doi: 10.1007/s40495-019-00171-y

[157] Forrester SJ Kikuchi DS Hernandes MS Xu Q Griendling KK. Reactive oxygen species in metabolic and inflammatory signaling. Circ Res (2018) 122:877–902. doi: 10.1161/CIRCRESAHA.117.311401 29700084 PMC5926825

[158] Lamouille S Xu J Derynck R. Molecular mechanisms of epithelial–mesenchymal transition. Nat Rev Mol Cell Biol (2014) 15:178–96. doi: 10.1038/nrm3758 PMC4240281 24556840

[159] Taki M Abiko K Ukita M Murakami R Yamanoi K Yamaguchi K. Tumor immune microenvironment during epithelial–mesenchymal transition. Clin Cancer Res (2021) 27:4669–79. doi: 10.1158/1078-0432.CCR-20-4459 33827891

[160] Zhang B Liu Z Hu X. Inhibiting cancer metastasis *via* targeting NAPDH oxidase 4. Biochem Pharmacol (2013) 86:253–66. doi: 10.1016/j.bcp.2013.05.011 23688500

[161] Boudreau HE Casterline BW Rada B Korzeniowska A Leto TL. Nox4 involvement in TGF-beta and SMAD3-driven induction of the epithelial-to-mesenchymal transition and migration of breast epithelial cells. Free Radical Biol Med (2012) 53:1489–99. doi: 10.1016/j.freeradbiomed.2012.06.016 PMC3448829 22728268

[162] Prasad S Gupta SC Tyagi AK. Reactive oxygen species (ROS) and cancer: Role of antioxidative nutraceuticals. Cancer Lett (2017) 387:95–105. doi: 10.1016/j.canlet.2016.03.042 27037062

[163] Tobar N Guerrero J Smith PC Martínez J. NOX4-dependent ROS production by stromal mammary cells modulates epithelial MCF-7 cell migration. Br J Cancer (2010) 103:1040–7. doi: 10.1038/sj.bjc.6605847 PMC2965862 20717118

[164] Liao Z Chua D Tan NS. Reactive oxygen species: a volatile driver of field cancerization and metastasis. Mol Cancer (2019) 18:65. doi: 10.1186/s12943-019-0961-y 30927919 PMC6441160

[165] Tobar N Villar V Santibanez JF. ROS-NFκB mediates TGF-β1-induced expression of urokinase-type plasminogen activator, matrix metalloproteinase-9 and cell invasion. Mol Cell Biochem (2010) 340:195–202. doi: 10.1007/s11010-010-0418-5 20204677

[166] Kamiya T Goto A Kurokawa E Hara H Adachi T. Cross talk mechanism among EMT, ROS, and histone acetylation in phorbol ester-treated human breast cancer MCF-7 cells. Oxid Med Cell Longevity (2016) 2016:e1284372. doi: 10.1155/2016/1284372 PMC4830742 27127545

[167] Shin DH Dier U Melendez JA Hempel N. Regulation of MMP-1 expression in response to hypoxia is dependent on the intracellular redox status of metastatic bladder cancer cells. Biochim Biophys Acta (BBA) - Mol Basis Dis (2015) 1852:2593–602. doi: 10.1016/j.bbadis.2015.09.001 PMC4615546 26343184

[168] Tong L Chuang C-C Wu S Zuo L. Reactive oxygen species in redox cancer therapy. Cancer Lett (2015) 367:18–25. doi: 10.1016/j.canlet.2015.07.008 26187782

[169] Liu Y Cui Y Shi M Zhang Q Wang Q Chen X. Deferoxamine promotes MDA-MB-231 cell migration and invasion through increased ROS-dependent HIF-1α accumulation. CPB (2014) 33:1036–46. doi: 10.1159/000358674 24732598

[170] Han X Sun S Zhao M Cheng X Chen G Lin S. Celastrol stimulates hypoxia-inducible factor-1 activity in tumor cells by initiating the ROS/Akt/p70S6K signaling pathway and enhancing hypoxia-inducible factor-1α protein synthesis. PloS One (2014) 9:e112470. doi: 10.1371/journal.pone.0112470 25383959 PMC4226555

[171] Brewer GJ. Anticopper therapy against cancer and diseases of inflammation and fibrosis. Drug Discovery Today (2005) 10:1103–9. doi: 10.1016/S1359-6446(05)03541-5 16182195

[172] Goodman VL Brewer GJ Merajver SD. Copper deficiency as an anti-cancer strategy. Endocrine-Related Cancer (2004) 11:255–63. doi: 10.1677/erc.0.0110255 15163301

[173] Gupte A Mumper RJ. Elevated copper and oxidative stress in cancer cells as a target for cancer treatment. Cancer Treat Rev (2009) 35:32–46. doi: 10.1016/j.ctrv.2008.07.004 18774652

[174] Rigiracciolo DC Scarpelli A Lappano R Pisano A Santolla MF De Marco P. Copper activates HIF-1α/GPER/VEGF signalling in cancer cells. Oncotarget (2015) 6:34158–77. doi: 10.18632/oncotarget.5779 PMC4741443 26415222

[175] Zhou X Yue GG-L Chan AM-L Tsui SK-W Fung K-P Sun H. A novel autophagy inducer, exerts anti-tumor activity through the suppression of Akt/mTOR/p70S6K signaling pathway in breast cancer. Biochem Pharmacol (2017) 142:58–70. doi: 10.1016/j.bcp.2017.06.133 28669564

[176] Ushio-Fukai M Nakamura Y. Reactive oxygen species and angiogenesis: NADPH oxidase as target for cancer therapy. Cancer Lett (2008) 266:37–52. doi: 10.1016/j.canlet.2008.02.044 18406051 PMC2673114

[177] Dewhirst MW Cao Y Moeller B. Cycling hypoxia and free radicals regulate angiogenesis and radiotherapy response. Nat Rev Cancer (2008) 8:425–37. doi: 10.1038/nrc2397 PMC3943205 18500244

[178] Ushio-Fukai M Alexander RW. Reactive oxygen species as mediators of angiogenesis signaling: role of NAD(P)H oxidase. Mol Cell Biochem (2004) 264:85–97. doi: 10.1023/b:mcbi.0000044378.09409.b5 15544038

[179] Giorgio M Migliaccio E Orsini F Paolucci D Moroni M Contursi C. Electron transfer between cytochrome c and p66Shc generates reactive oxygen species that trigger mitochondrial apoptosis. Cell (2005) 122:221–33. doi: 10.1016/j.cell.2005.05.011 16051147

[180] Danial NN Korsmeyer SJ. Cell death: Critical control points. Cell (2004) 116:205–19. doi: 10.1016/S0092-8674(04)00046-7 14744432

[181] Simon H-U Haj-Yehia A Levi-Schaffer F. Role of reactive oxygen species (ROS) in apoptosis induction. Apoptosis (2000) 5:415–8. doi: 10.1023/A:1009616228304 11256882

[182] Redza-Dutordoir M Averill-Bates DA. Activation of apoptosis signalling pathways by reactive oxygen species. Biochim Biophys Acta (2016) 1863:2977–92. doi: 10.1016/j.bbamcr.2016.09.012 27646922

[183] Woo CC Hsu A Kumar AP Sethi G Tan KHB. Thymoquinone inhibits tumor growth and induces apoptosis in a breast cancer xenograft mouse model: The role of p38 MAPK and ROS. PloS One (2013) 8:e75356. doi: 10.1371/journal.pone.0075356 24098377 PMC3788809

[184] Dai X Wang L Deivasigamni A Looi CY Karthikeyan C Trivedi P. A novel benzimidazole derivative, MBIC inhibits tumor growth and promotes apoptosis *via* activation of ROS-dependent JNK signaling pathway in hepatocellular carcinoma. Oncotarget (2017) 8:12831–42. doi: 10.18632/oncotarget.14606 PMC5355059 28086233

[185] Kim C Lee S-G Yang WM Arfuso F Um J-Y Kumar AP. Formononetin-induced oxidative stress abrogates the activation of STAT3/5 signaling axis and suppresses the tumor growth in multiple myeloma preclinical model. Cancer Lett (2018) 431:123–41. doi: 10.1016/j.canlet.2018.05.038 29857127

[186] Khan M Chen H Wan X Tania M Xu A Chen F. Regulatory effects of resveratrol on antioxidant enzymes: A mechanism of growth inhibition and apoptosis induction in cancer cells. Mol Cells (2013) 35:219–25. doi: 10.1007/s10059-013-2259-z PMC3887918 23456297

[187] Eddy SR. Non–coding RNA genes and the modern RNA world. Nat Rev Genet (2001) 2:919–29. doi: 10.1038/35103511 11733745

[188] Zhang P Wu W Chen Q Chen M. Non-coding RNAs and their integrated networks. J Integr Bioinform (2019) 16:1–12. doi: 10.1515/jib-2019-0027 PMC6798851 31301674

[189] Korpal M Ell BJ Buffa FM Ibrahim T Blanco MA Celià-Terrassa T. Direct targeting of Sec23a by miR-200s influences cancer cell secretome and promotes metastatic colonization. Nat Med (2011) 17:1101–8. doi: 10.1038/nm.2401 PMC3169707 21822286

[190] Mizuno T Chou MY Inouye M. A unique mechanism regulating gene expression: translational inhibition by a complementary RNA transcript (micRNA). Proc Natl Acad Sci U.S.A. (1984) 81:1966–70. doi: 10.1073/pnas.81.7.1966 PMC345417 6201848

[191] Delihas N. Discovery and characterization of the first non-coding RNA that regulates gene expression, micF RNA: A historical perspective. World J Biol Chem (2015) 6:272–80. doi: 10.4331/wjbc.v6.i4.272 PMC4657122 26629310

[192] Dahariya S Paddibhatla I Kumar S Raghuwanshi S Pallepati A Gutti RK. Long non-coding RNA: Classification, biogenesis and functions in blood cells. Mol Immunol (2019) 112:82–92. doi: 10.1016/j.molimm.2019.04.011 31079005

[193] Losko M Kotlinowski J Jura J. Long noncoding RNAs in metabolic syndrome related disorders. Mediators Inflammation (2016) 2016:e5365209. doi: 10.1155/2016/5365209 PMC5110871 27881904

[194] Su Y Wu H Pavlosky A Zou L-L Deng X Zhang Z-X. Regulatory non-coding RNA: new instruments in the orchestration of cell death. Cell Death Dis (2016) 7:e2333–3. doi: 10.1038/cddis.2016.210 PMC5108314 27512954

[195] Zheng M Wu Z Wu A Huang Z He N Xie X. MiR-145 promotes TNF-α-induced apoptosis by facilitating the formation of RIP1-FADDcaspase-8 complex in triple-negative breast cancer. Tumour Biol (2016) 37:8599–607. doi: 10.1007/s13277-015-4631-4 26733177

[196] Mitsunaga S Ikeda M Shimizu S Ohno I Furuse J Inagaki M. Serum levels of IL-6 and IL-1β can predict the efficacy of gemcitabine in patients with advanced pancreatic cancer. Br J Cancer (2013) 108:2063–9. doi: 10.1038/bjc.2013.174 PMC3670479 23591198

[197] Gong C Qu S Liu B Pan S Jiao Y Nie Y. MiR-106b expression determines the proliferation paradox of TGF-β in breast cancer cells. Oncogene (2015) 34:84–93. doi: 10.1038/onc.2013.525 24292682

[198] Chen L Deng H Cui H Fang J Zuo Z Deng J. Inflammatory responses and inflammation-associated diseases in organs. Oncotarget (2017) 9:7204–18. doi: 10.18632/oncotarget.23208 PMC5805548 29467962

[199] O’Connell RM Rao DS Baltimore D. microRNA regulation of inflammatory responses. Annu Rev Immunol (2012) 30:295–312. doi: 10.1146/annurev-immunol-020711-075013 22224773

[200] O’Neill LA Sheedy FJ McCoy CE. MicroRNAs: the fine-tuners of toll-like receptor signalling. Nat Rev Immunol (2011) 11:163–75. doi: 10.1038/nri2957 21331081

[201] Nejad C Stunden HJ Gantier MP. A guide to miRNAs in inflammation and innate immune responses. FEBS J (2018) 285:3695–716. doi: 10.1111/febs.14482 29688631

[202] O’Connell RM Rao DS Chaudhuri AA Baltimore D. Physiological and pathological roles for microRNAs in the immune system. Nat Rev Immunol (2010) 10:111–22. doi: 10.1038/nri2708 20098459

[203] Taganov KD Boldin MP Baltimore D. MicroRNAs and immunity: Tiny players in a big field. Immunity (2007) 26:133–7. doi: 10.1016/j.immuni.2007.02.005 17307699

[204] Wu Z Lu H Sheng J Li L. Inductive microRNA-21 impairs anti-mycobacterial responses by targeting IL-12 and bcl-2. FEBS Lett (2012) 586:2459–67. doi: 10.1016/j.febslet.2012.06.004 22710123

[205] Taganov KD Boldin MP Chang K-J Baltimore D. NF-kappaB-dependent induction of microRNA miR-146, an inhibitor targeted to signaling proteins of innate immune responses. Proc Natl Acad Sci U.S.A. (2006) 103:12481–6. doi: 10.1073/pnas.0605298103 PMC1567904 16885212

[206] Ishii H Vodnala SK Achyut BR So JY Hollander MC Greten TF. miR-130a and miR-145 reprogram gr-1+CD11b+ myeloid cells and inhibit tumor metastasis through improved host immunity. Nat Commun (2018) 9:2611. doi: 10.1038/s41467-018-05023-9 29973593 PMC6031699

[207] Chiodoni C Cancila V Renzi TA Perrone M Tomirotti AM Sangaletti S. Transcriptional profiles and stromal changes reveal bone marrow adaptation to early breast cancer in association with deregulated circulating microRNAs. Cancer Res (2020) 80:484–98. doi: 10.1158/0008-5472.CAN-19-1425 31776132

[208] Qin Z Kearney P Plaisance K Parsons CH. Pivotal advance: Kaposi’s sarcoma-associated herpesvirus (KSHV)-encoded microRNA specifically induce IL-6 and IL-10 secretion by macrophages and monocytes. J Leukoc Biol (2010) 87:25–34. doi: 10.1189/jlb.0409251 20052801 PMC2801620

[209] Seif F Vaseghi H Ariana M Ganji SM Nazari M Rad KK. Overexpression of miR-490-5p/miR-490-3p potentially induces IL-17-Producing T cells in patients with breast cancer. Eur J Breast Health (2022) 18:141–7. doi: 10.4274/ejbh.galenos.2022.2021-10-4 PMC8987853 35445179

[210] Soheilifar MH Vaseghi H Seif F Ariana M Ghorbanifar S Habibi N. Concomitant overexpression of mir-182-5p and mir-182-3p raises the possibility of IL-17-producing treg formation in breast cancer by targeting CD3d, ITK, FOXO1, and NFATs: A meta-analysis and experimental study. Cancer Sci (2021) 112:589–603. doi: 10.1111/cas.14764 33283362 PMC7893989

[211] Kim S Lee ES ji LE Jung JY Lee SB Lee HJ. Targeted eicosanoids profiling reveals a prostaglandin reprogramming in breast cancer by microRNA-155. J Exp Clin Cancer Res (2021) 40:43. doi: 10.1186/s13046-021-01839-4 33494773 PMC7831268

[212] Majumder M Landman E Liu L Hess D Lala PK. COX-2 elevates oncogenic miR-526b in breast cancer by EP4 activation. Mol Cancer Res (2015) 13:1022–33. doi: 10.1158/1541-7786.MCR-14-0543 25733698

[213] Majumder M Dunn L Liu L Hasan A Vincent K Brackstone M. COX-2 induces oncogenic micro RNA miR655 in human breast cancer. Sci Rep (2018) 8:327. doi: 10.1038/s41598-017-18612-3 29321644 PMC5762661

[214] Majumder M Nandi P Omar A Ugwuagbo KC Lala PK. EP4 as a therapeutic target for aggressive human breast cancer. Int J Mol Sci (2018) 19:1019. doi: 10.3390/ijms19041019 PMC5979567 29596308

[215] Hunter S Nault B Ugwuagbo KC Maiti S Majumder M. Mir526b and Mir655 promote tumour associated angiogenesis and lymphangiogenesis in breast cancer. Cancers (Basel) (2019) 11:938. doi: 10.3390/cancers11070938 PMC6678879 31277414

[216] De Paz Linares GA Opperman RM Majumder M Lala PK. Prostaglandin E2 receptor 4 (EP4) as a therapeutic target to impede breast cancer-associated angiogenesis and lymphangiogenesis. Cancers (Basel) (2021) 13:942. doi: 10.3390/cancers13050942 33668160 PMC7956318

[217] Rezaei Z Sadri F. MicroRNAs involved in inflammatory breast cancer: Oncogene and tumor suppressors with possible targets. DNA Cell Biol (2021) 40:499–512. doi: 10.1089/dna.2020.6320 33493414

[218] Germano G Allavena P Mantovani A. Cytokines as a key component of cancer-related inflammation. Cytokine (2008) 43:374–9. doi: 10.1016/j.cyto.2008.07.014 18701317

[219] Hirschberger S Hinske LC Kreth S. MiRNAs: dynamic regulators of immune cell functions in inflammation and cancer. Cancer Lett (2018) 431:11–21. doi: 10.1016/j.canlet.2018.05.020 29800684

[220] Moi P Chan K Asunis I Cao A Kan YW. Isolation of NF-E2-related factor 2 (Nrf2), a NF-E2-like basic leucine zipper transcriptional activator that binds to the tandem NF-E2/AP1 repeat of the beta-globin locus control region. Proc Natl Acad Sci U.S.A. (1994) 91:9926–30. doi: 10.1073/pnas.91.21.9926 PMC44930 7937919

[221] Wu S Lu H Bai Y. Nrf2 in cancers: A double-edged sword. Cancer Med (2019) 8:2252–67. doi: 10.1002/cam4.2101 PMC6536957 30929309

[222] Taguchi K Yamamoto M. The KEAP1–NRF2 system in cancer. Front Oncol (2017) 7:85. doi: 10.3389/fonc.2017.00085 28523248 PMC5415577

[223] Bellezza I Giambanco I Minelli A Donato R. Nrf2-Keap1 signaling in oxidative and reductive stress. Biochim Biophys Acta Mol Cell Res (2018) 1865:721–33. doi: 10.1016/j.bbamcr.2018.02.010 29499228

[224] Itoh K Mimura J Yamamoto M. Discovery of the negative regulator of Nrf2, Keap1: A historical overview. Antioxidants Redox Signaling (2010) 13:1665–78. doi: 10.1089/ars.2010.3222 20446768

[225] Ayers D Baron B Hunter T. miRNA influences in NRF2 pathway interactions within cancer models. J Nucleic Acids (2015) 2015:143636. doi: 10.1155/2015/143636 26345522 PMC4546755

[226] Singh B Ronghe AM Chatterjee A Bhat NK Bhat HK. MicroRNA-93 regulates NRF2 expression and is associated with breast carcinogenesis. Carcinogenesis (2013) 34:1165–72. doi: 10.1093/carcin/bgt026 PMC3643421 23492819

[227] Yang M Yao Y Eades G Zhang Y Zhou Q. MiR-28 regulates Nrf2 expression through a Keap1-independent mechanism. Breast Cancer Res Treat (2011) 129:983–91. doi: 10.1007/s10549-011-1604-1 PMC3752913 21638050

[228] Eades G Yang M Yao Y Zhang Y Zhou Q. miR-200a regulates Nrf2 activation by targeting Keap1 mRNA in breast cancer cells. J Biol Chem (2011) 286:40725–33. doi: 10.1074/jbc.M111.275495 PMC3220489 21926171

[229] Yi J Huang W-Z Wen Y-Q Yi Y-C. Effect of miR-101 on proliferation and oxidative stress-induced apoptosis of breast cancer cells *via* Nrf2 signaling pathway. Eur Rev Med Pharmacol Sci (2019) 23:8931–9. doi: 10.26355/eurrev_201910_19291 31696480

[230] Wang B Teng Y Liu Q. MicroRNA-153 regulates NRF2 expression and is associated with breast carcinogenesis. Clin Lab (2016) 62:39–47. doi: 10.7754/clin.lab.2015.150518 27012032

[231] Keklikoglou I Koerner C Schmidt C Zhang JD Heckmann D Shavinskaya A. MicroRNA-520/373 family functions as a tumor suppressor in estrogen receptor negative breast cancer by targeting NF-κB and TGF-β signaling pathways. Oncogene (2012) 31:4150–63. doi: 10.1038/onc.2011.571 22158050

[232] Körner C Keklikoglou I Bender C Wörner A Münstermann E Wiemann S. MicroRNA-31 sensitizes human breast cells to apoptosis by direct targeting of protein kinase c epsilon (PKCepsilon). J Biol Chem (2013) 288:8750–61. doi: 10.1074/jbc.M112.414128 PMC3605692 23364795

[233] Shukla K Sharma AK Ward A Will R Hielscher T Balwierz A. MicroRNA-30c-2-3p negatively regulates NF-κB signaling and cell cycle progression through downregulation of TRADD and CCNE1 in breast cancer. Mol Oncol (2015) 9:1106–19. doi: 10.1016/j.molonc.2015.01.008 PMC5528752 25732226

[234] Bott A Erdem N Lerrer S Hotz-Wagenblatt A Breunig C Abnaof K. miRNA-1246 induces pro-inflammatory responses in mesenchymal stem/stromal cells by regulating PKA and PP2A. Oncotarget (2017) 8:43897–914. doi: 10.18632/oncotarget.14915 PMC5546423 28159925

[235] Li B Lu Y Yu L Han X Wang H Mao J. miR-221/222 promote cancer stem-like cell properties and tumor growth of breast cancer *via* targeting PTEN and sustained Akt/NF-κB/COX-2 activation. Chem Biol Interact (2017) 277:33–42. doi: 10.1016/j.cbi.2017.08.014 28844858

[236] Singh R Yadav V Kumar S Saini N. MicroRNA-195 inhibits proliferation, invasion and metastasis in breast cancer cells by targeting FASN, HMGCR, ACACA and CYP27B1. Sci Rep (2015) 5:1–15. doi: 10.1038/srep17454 PMC4668367 26632252

[237] Lou C Xiao M Cheng S Lu X Jia S Ren Y. MiR-485-3p and miR-485-5p suppress breast cancer cell metastasis by inhibiting PGC-1 α expression. Cell Death Dis (2016) 7:e2159–9. doi: 10.1038/cddis.2016.27 PMC4823935 27010860

[238] Eastlack SC Dong S Ivan C Alahari SK. Suppression of PDHX by microRNA-27b deregulates cell metabolism and promotes growth in breast cancer. Mol Cancer (2018) 17:100. doi: 10.1186/s12943-018-0851-8 30012170 PMC6048708

[239] Peng L-N Deng X-Y Gan X-X Zhang J-H Ren G-H Shen F. Targeting of TLE3 by miR-3677 in human breast cancer promotes cell proliferation, migration and invasion. Oncol Lett (2020) 19:1409–17. doi: 10.3892/ol.2019.11241 PMC6960393 32002031

[240] Sripada L Singh K Lipatova AV Singh A Prajapati P Tomar D. Hsa-miR-4485 regulates mitochondrial functions and inhibits the tumorigenicity of breast cancer cells. J Mol Med (Berl) (2017) 95:641–51. doi: 10.1007/s00109-017-1517-5 28220193

[241] Loss of function of miR-342-3p results in MCT1 over-expression and contributes to oncogenic metabolic reprogramming in triple negative breast cancer. Available at: https://www.nature.com/articles/s41598-018-29708-9 (Accessed August 22, 2022). 10.1038/s41598-018-29708-9 PMC6095912 30115973

[242] Jiang S Zhang L-F Zhang H-W Hu S Lu M-H Liang S. A novel miR-155/miR-143 cascade controls glycolysis by regulating hexokinase 2 in breast cancer cells. EMBO J (2012) 31:1985–98. doi: 10.1038/emboj.2012.45 PMC3343331 22354042

[243] Bs H Hs R NK J K EL H M Kh K Ms J. Tumor suppressor miRNA-204-5p regulates growth, metastasis, and immune microenvironment remodeling in breast cancer. Cancer Res (2019) 79:1520–34. doi: 10.1158/0008-5472.CAN-18-0891 30737233

[244] Liang H Xiao J Zhou Z Wu J Ge F Li Z. Hypoxia induces miR-153 through the IRE1α-XBP1 pathway to fine tune the HIF1α/VEGFA axis in breast cancer angiogenesis. Oncogene (2018) 37:1961–75. doi: 10.1038/s41388-017-0089-8 PMC5895606 29367761

[245] Nagpal N Ahmad HM Chameettachal S Sundar D Ghosh S Kulshreshtha R. HIF-inducible miR-191 promotes migration in breast cancer through complex regulation of TGFβ-signaling in hypoxic microenvironment. Sci Rep (2015) 5:9650. doi: 10.1038/srep09650 25867965 PMC4394754

[246] Krutilina R Sun W Sethuraman A Brown M Seagroves TN Pfeffer LM. MicroRNA-18a inhibits hypoxia-inducible factor 1α activity and lung metastasis in basal breast cancers. Breast Cancer Res (2014) 16:R78. doi: 10.1186/bcr3693 25069832 PMC4405876

[247] Wu Z Cai X Huang C Xu J Liu A. miR-497 suppresses angiogenesis in breast carcinoma by targeting HIF-1α. Oncol Rep (2016) 35:1696–702. doi: 10.3892/or.2015.4529 26718330

[248] Song Y-H Wang J Nie G Chen Y-J Li X Jiang X. MicroRNA-509-5p functions as an anti-oncogene in breast cancer *via* targeting SOD2. Eur Rev Med Pharmacol Sci (2017) 21:3617–25. 28925482

[249] Degli Esposti D Aushev VN Lee E Cros M-P Zhu J Herceg Z. miR-500a-5p regulates oxidative stress response genes in breast cancer and predicts cancer survival. Sci Rep (2017) 7:15966. doi: 10.1038/s41598-017-16226-3 29162888 PMC5698490

[250] Pajic M Froio D Daly S Doculara L Millar E Graham PH. miR-139-5p modulates radiotherapy resistance in breast cancer by repressing multiple gene networks of DNA repair and ROS defense. Cancer Res (2018) 78:501–15. doi: 10.1158/0008-5472.CAN-16-3105 29180477

[251] Shin B Feser R Nault B Hunter S Maiti S Ugwuagbo KC. miR526b and miR655 induce oxidative stress in breast cancer. Int J Mol Sci (2019) 20:4039. doi: 10.3390/ijms20164039 PMC6720387 31430859

[252] Simone NL Soule BP Ly D Saleh AD Savage JE Degraff W. Ionizing radiation-induced oxidative stress alters miRNA expression. PloS One (2009) 4:e6377. doi: 10.1371/journal.pone.0006377 19633716 PMC2712071

[253] Leisegang MS Schröder K Brandes RP. Redox regulation and noncoding RNAs. Antioxidants Redox Signaling (2017) 29:793–812. doi: 10.1089/ars.2017.7276 28816061

[254] Lu C Zhou D Wang Q Liu W Yu F Wu F. Crosstalk of MicroRNAs and oxidative stress in the pathogenesis of cancer. Oxid Med Cell Longev (2020) 2020:1–13. doi: 10.1155/2020/2415324 PMC7204110 32411322

[255] Carbonell T Gomes AV. MicroRNAs in the regulation of cellular redox status and its implications in myocardial ischemia-reperfusion injury. Redox Biol (2020) 36:101607. doi: 10.1016/j.redox.2020.101607 32593128 PMC7322687

[256] Weitz SH Gong M Barr I Weiss S Guo F. Processing of microRNA primary transcripts requires heme in mammalian cells. PNAS (2014) 111:1861–6. doi: 10.1073/pnas.1309915111 PMC3918773 24449907

[257] Cheng X Ku C-H Siow RCM. Regulation of the Nrf2 antioxidant pathway by microRNAs: New players in micromanaging redox homeostasis. Free Radic Biol Med (2013) 64:4–11. doi: 10.1016/j.freeradbiomed.2013.07.025 23880293

[258] Mori MA Raghavan P Thomou T Boucher J Robida-Stubbs S Macotela Y. Role of microRNA processing in adipose tissue in stress defense and longevity. Cell Metab (2012) 16:336–47. doi: 10.1016/j.cmet.2012.07.017 PMC3461823 22958919

[259] Ungvari Z Tucsek Z Sosnowska D Toth P Gautam T Podlutsky A. Aging-induced dysregulation of dicer1-dependent microRNA expression impairs angiogenic capacity of rat cerebromicrovascular endothelial cells. J Gerontol A Biol Sci Med Sci (2013) 68:877–91. doi: 10.1093/gerona/gls242 PMC3712357 23239824

[260] Shilo S Roy S Khanna S Sen CK. Evidence for the involvement of miRNA in redox regulated angiogenic response of human microvascular endothelial cells. Arterioscler Thromb Vasc Biol (2008) 28:471–7. doi: 10.1161/ATVBAHA.107.160655 18258815

[261] Wang J-X Gao J Ding S-L Wang K Jiao J-Q Wang Y. Oxidative modification of miR-184 enables it to target bcl-xL and bcl-w. Mol Cell (2015) 59:50–61. doi: 10.1016/j.molcel.2015.05.003 26028536

[262] Xu Q Liu L-Z Yin Y He J Li Q Qian X. Regulatory circuit of PKM2/NF-κB/miR-148a/152-modulated tumor angiogenesis and cancer progression. Oncogene (2015) 34:5482–93. doi: 10.1038/onc.2015.6 25703326

[263] Ahir M Bhattacharya S Karmakar S Mukhopadhyay A Mukherjee S Ghosh S. Tailored-CuO-nanowire decorated with folic acid mediated coupling of the mitochondrial-ROS generation and miR425-PTEN axis in furnishing potent anti-cancer activity in human triple negative breast carcinoma cells. Biomaterials (2016) 76:115–32. doi: 10.1016/j.biomaterials.2015.10.044 26520043

[264] Guo Y Li P Bledsoe G Yang Z-R Chao L Chao J. Kallistatin inhibits TGF-β-induced endothelial-mesenchymal transition by differential regulation of microRNA-21 and eNOS expression. Exp Cell Res (2015) 337:103–10. doi: 10.1016/j.yexcr.2015.06.021 PMC4560618 26156753

[265] Dai X Fang M Li S Yan Y Zhong Y Du B. miR-21 is involved in transforming growth factor β1-induced chemoresistance and invasion by targeting PTEN in breast cancer. Oncol Lett (2017) 14:6929–36. doi: 10.3892/ol.2017.7007 PMC5678423 29151919

[266] Chao J Guo Y Li P Chao L. Role of kallistatin treatment in aging and cancer by modulating miR-34a and miR-21 expression. Oxid Med Cell Longev (2017) 2017:5025610. doi: 10.1155/2017/5025610 28744338 PMC5506461

[267] Grønbaek K Hother C Jones PA. Epigenetic changes in cancer. APMIS (2007) 115:1039–59. doi: 10.1111/j.1600-0463.2007.apm_636.xml.x 18042143

[268] Han L Witmer PDW Casey E Valle D Sukumar S. DNA Methylation regulates microRNA expression. Cancer Biol Ther (2007) 6:1290–4. doi: 10.4161/cbt.6.8.4486 17660710

[269] Aure MR Fleischer T Bjørklund S Ankill J Castro-Mondragon JA OSBREAC. Crosstalk between microRNA expression and DNA methylation drives the hormone-dependent phenotype of breast cancer. Genome Med (2021) 13:72. doi: 10.1186/s13073-021-00880-4 33926515 PMC8086068

[270] Druz A Betenbaugh M Shiloach J. Glucose depletion activates mmu-miR-466h-5p expression through oxidative stress and inhibition of histone deacetylation. Nucleic Acids Res (2012) 40:7291–302. doi: 10.1093/nar/gks452 PMC3424575 22638581

[271] Singh A Happel C Manna SK Acquaah-Mensah G Carrerero J Kumar S. Transcription factor NRF2 regulates miR-1 and miR-206 to drive tumorigenesis. J Clin Invest (2013) 123:2921–34. doi: 10.1172/JCI66353 PMC3696551 23921124

[272] Druz A Chu C Majors B Santuary R Betenbaugh M Shiloach J. A novel microRNA mmu-miR-466h affects apoptosis regulation in mammalian cells. Biotechnol Bioeng (2011) 108:1651–61. doi: 10.1002/bit.23092 PMC3099433 21337326

[273] Cosentino G Plantamura I Cataldo A Iorio MV. MicroRNA and oxidative stress interplay in the context of breast cancer pathogenesis. Int J Mol Sci (2019) 20:275–84. doi: 10.3390/ijms20205143 PMC6829356 31627322

[274] Thorsen SB Obad S Jensen NF Stenvang J Kauppinen S. The therapeutic potential of microRNAs in cancer. Cancer J (2012) 18:275–84. doi: 10.1097/PPO.0b013e318258b5d6 22647365

[275] He J Jiang B-H. Interplay between reactive oxygen species and MicroRNAs in cancer. Curr Pharmacol Rep (2016) 2:82–90. doi: 10.1007/s40495-016-0051-4 27284501 PMC4894750

[276] Gorrini C Baniasadi PS Harris IS Silvester J Inoue S Snow B. BRCA1 interacts with Nrf2 to regulate antioxidant signaling and cell survival. J Exp Med (2013) 210:1529–44. doi: 10.1084/jem.20121337 PMC3727320 23857982

[277] Temiz E Koyuncu İ Sahin E. CCT3 suppression prompts apoptotic machinery through oxidative stress and energy deprivation in breast and prostate cancers. Free Radic Biol Med (2021) 165:88–99. doi: 10.1016/j.freeradbiomed.2021.01.016 33508424

[278] Shang M Wu Y Wang Y Cai Y Jin J Yang Z. Dual antisense oligonucleotide targeting miR-21/miR-155 synergize photodynamic therapy to treat triple-negative breast cancer and inhibit metastasis. BioMed Pharmacother (2022) 146:112564. doi: 10.1016/j.biopha.2021.112564 34954643

[279] Cataldo A Cheung DG Balsari A Tagliabue E Coppola V Iorio MV. miR-302b enhances breast cancer cell sensitivity to cisplatin by regulating E2F1 and the cellular DNA damage response. Oncotarget (2015) 7:786–97. doi: 10.18632/oncotarget.6381 PMC4808033 26623722

[280] Hu G Zhao X Wang J Lv L Wang C Feng L. miR-125b regulates the drug-resistance of breast cancer cells to doxorubicin by targeting HAX-1. Oncol Lett (2018) 15:1621–9. doi: 10.3892/ol.2017.7476 PMC5774474 29434858

[281] Roscigno G Puoti I Giordano I Donnarumma E Russo V Affinito A. MiR-24 induces chemotherapy resistance and hypoxic advantage in breast cancer. Oncotarget (2017) 8:19507–21. doi: 10.18632/oncotarget.14470 PMC5386701 28061479

[282] Luo M Ding L Li Q Yao H. miR-668 enhances the radioresistance of human breast cancer cell by targeting IκBα. Breast Cancer (2017) 24:673–82. doi: 10.1007/s12282-017-0756-1 28138801

[283] Sun X Li Y Zheng M Zuo W Zheng W. MicroRNA-223 increases the sensitivity of triple-negative breast cancer stem cells to TRAIL-induced apoptosis by targeting HAX-1. PLoS One (2016) 11:e0162754. doi: 10.1371/journal.pone.0162754 27618431 PMC5019415

[284] Yadav P Sharma P Sundaram S Venkatraman G Bera AK Karunagaran D. SLC7A11/xCT is a target of miR-5096 and its restoration partially rescues miR-5096-mediated ferroptosis and anti-tumor effects in human breast cancer cells. Cancer Lett (2021) 522:211–24. doi: 10.1016/j.canlet.2021.09.033 34571083

[285] Wu Z-H Tang Y Yu H Li H-D. The role of ferroptosis in breast cancer patients: a comprehensive analysis. Cell Death Discovery (2021) 7:93. doi: 10.1038/s41420-021-00473-5 33947836 PMC8097021

